# Supplementary figures and images for: The Interaction between Sleep and Development on Wake EEG Oscillations
Source: eNeuro. 2026 Apr 21;13(4):ENEURO.0384-25.2026. doi: 10.1523/ENEURO.0384-25.2026 (PMC13132015; doi:10.1523/ENEURO.0384-25.2026)

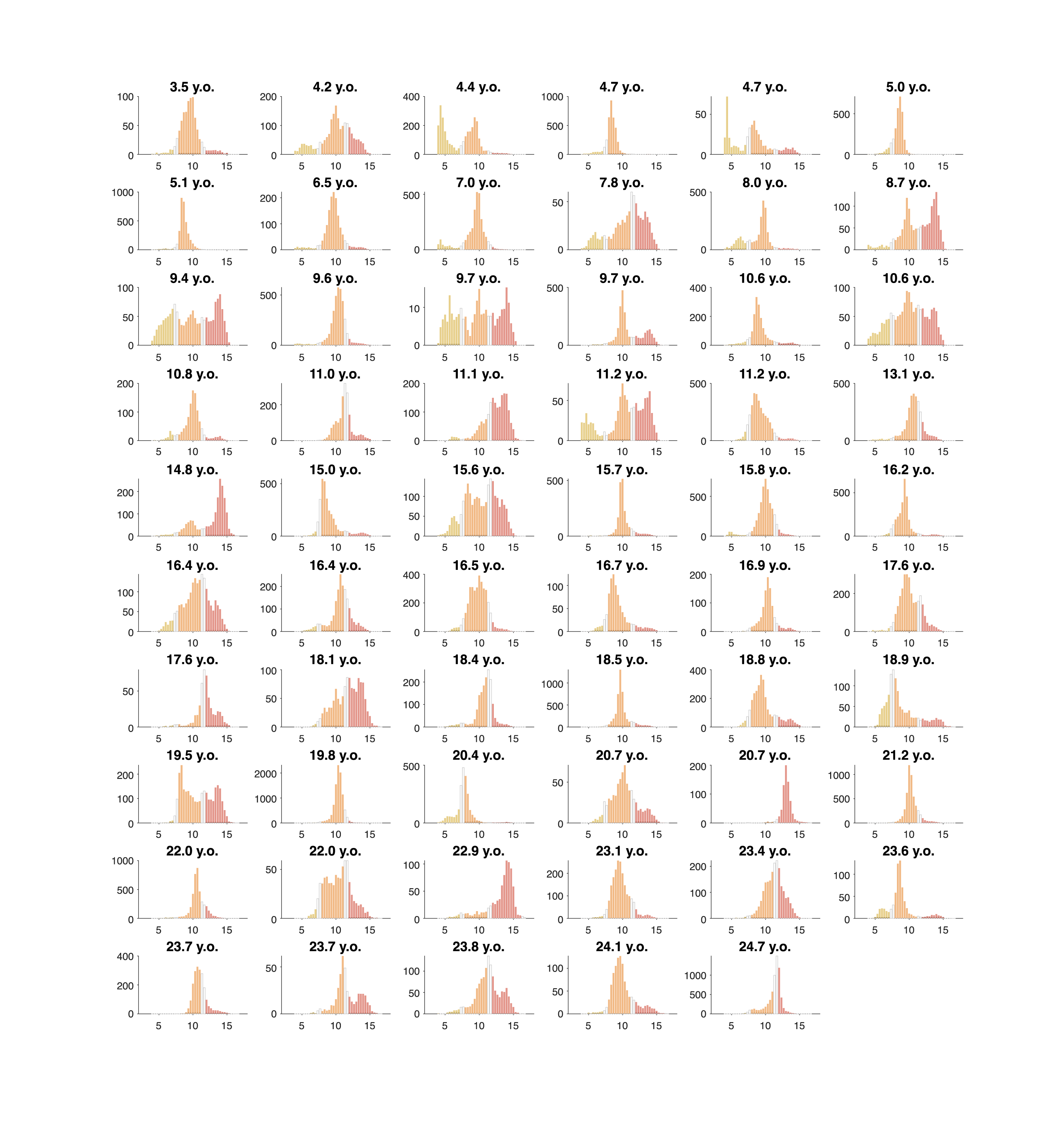

Supplement: Figure 1-1 — Individuals’ distribution of oscillation densities by frequency. Each plot is from one participant, with frequency (Hz) on the x-axis and density (% recording) on the y-axis. Colors reflect the frequency bands used in Main Figure 6 and Figure 7: yellow for theta (4-7 Hz); orange alpha (8-11 Hz); red low beta (12-16 Hz). This figure only includes evening oddball recordings from neurotypical participants, sorted by age. Download Figure 1-1, TIF file. [file eneuro-13-ENEURO.0384-25.2026-s002.tif]

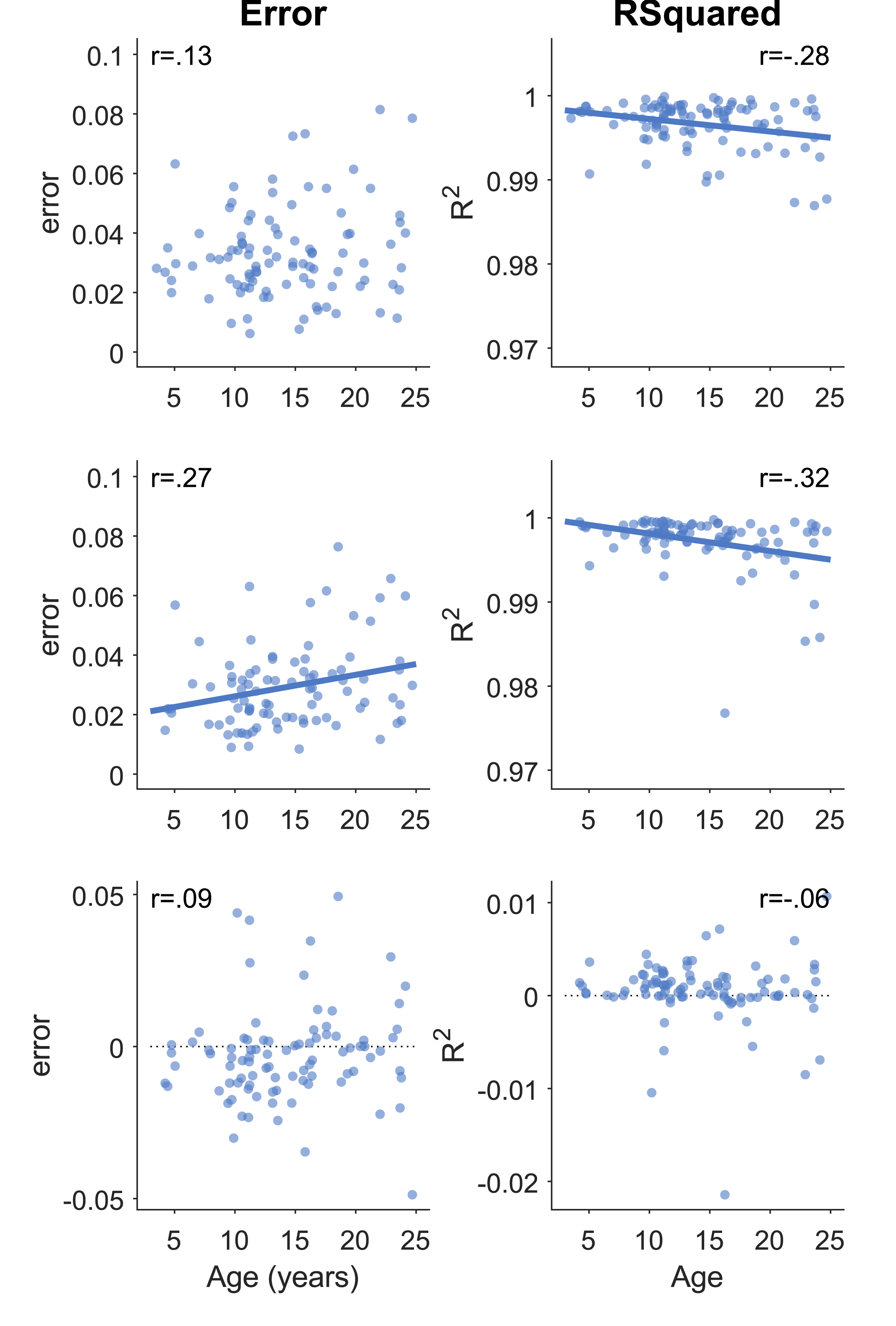

Supplement: Figure 3-1 — Specparam fitting estimates correlated with age. Only auditory oddball recordings are included, pooling both neurotypical and ADHD participants, the same as in Main Figure 3. Each dot represents a single participant. For participants with multiple sessions, values across sessions were first averaged. Pearson’s correlations were done for each figure, with r values provided in the corner. If the p-value was less than .05, a correlation line was drawn (without correcting for multiple comparisons). The same linear mixed effects model was applied to the fitting estimates of the specparam model, to evaluate whether any potential differences in model fitting could explain the aperiodic results. Mean absolute errors had a trending effect of age (beta = 0.000, t = 1.69, p = .091, df = 1234), curiously increasing with age, and a significant decrease the morning after sleep (beta = -0.007, t = -3.75, p < .001, df = 1234). There was no significant effect of ADHD (beta = 0.001, t = 0.36, p = .717, df = 1234) or sex (beta = 0.000, t = 0.09, p = .930, df = 1234), and the Time * Age interaction was only trending (beta = 0.000, t = 1.75, p = .080, df = 1234). R-squared values significantly decreased with age (beta = -0.000, t = -3.72, p < .001, df = 1234), significant increased after sleep (beta = 0.001, t = 2.96, p = .003, df = 1234), had no significant effect of ADHD (beta = -0.000, t = -0.87, p = .384, df = 1234), sex (beta = -0.000, t = -0.47, p = .639, df = 1234), or Time * Age interaction (beta = -0.000, t = -0.90, p = .366, df = 1234). Given that model fitting varied systematically with the factors of interest, it is possible some of the effects observed for aperiodic exponents and offsets are attributable to differences in model fitting. However, the main effects of aperiodic exponents and offsets were substantially larger than these effects of the model fits. Download Figure 3-1, TIF file. [file eneuro-13-ENEURO.0384-25.2026-s003.tif]

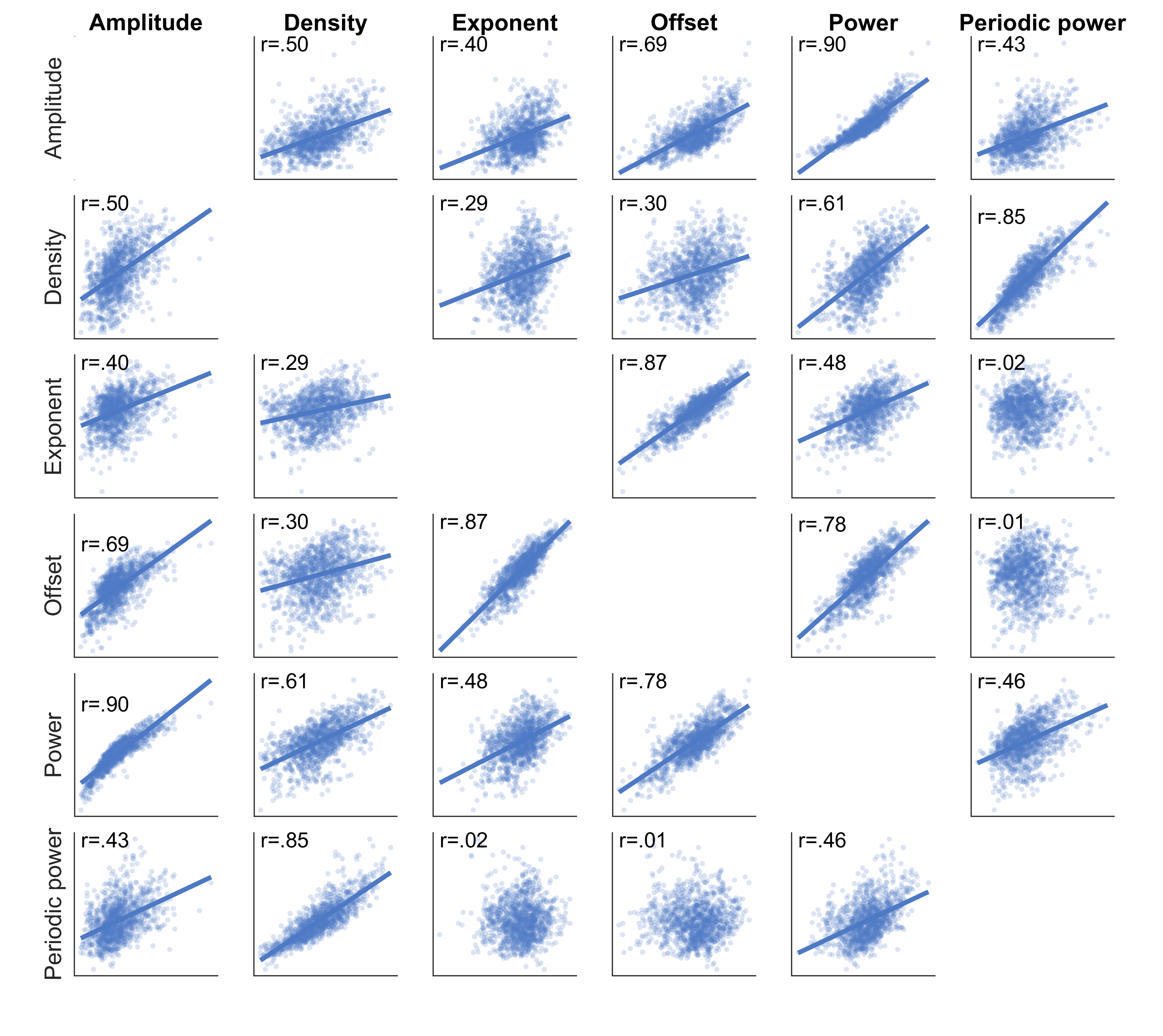

Supplement: Figure 3-2 — Correlations between outcome measures. Each dot represents the data of a recording (n = 1243). Pearson’s r values are provided correlating all measures from both datasets, and significant correlations (p-value < .05, FDR corrected for multiple comparisons) have linear fits plotted separately for each dataset. Given that there are repeated measures from the same participants, these correlations violate assumptions of independence, but were conducted nevertheless to provide a simple metric to compare the relationship between different measures. Download Figure 3-2, TIF file. [file eneuro-13-ENEURO.0384-25.2026-s004.tif]

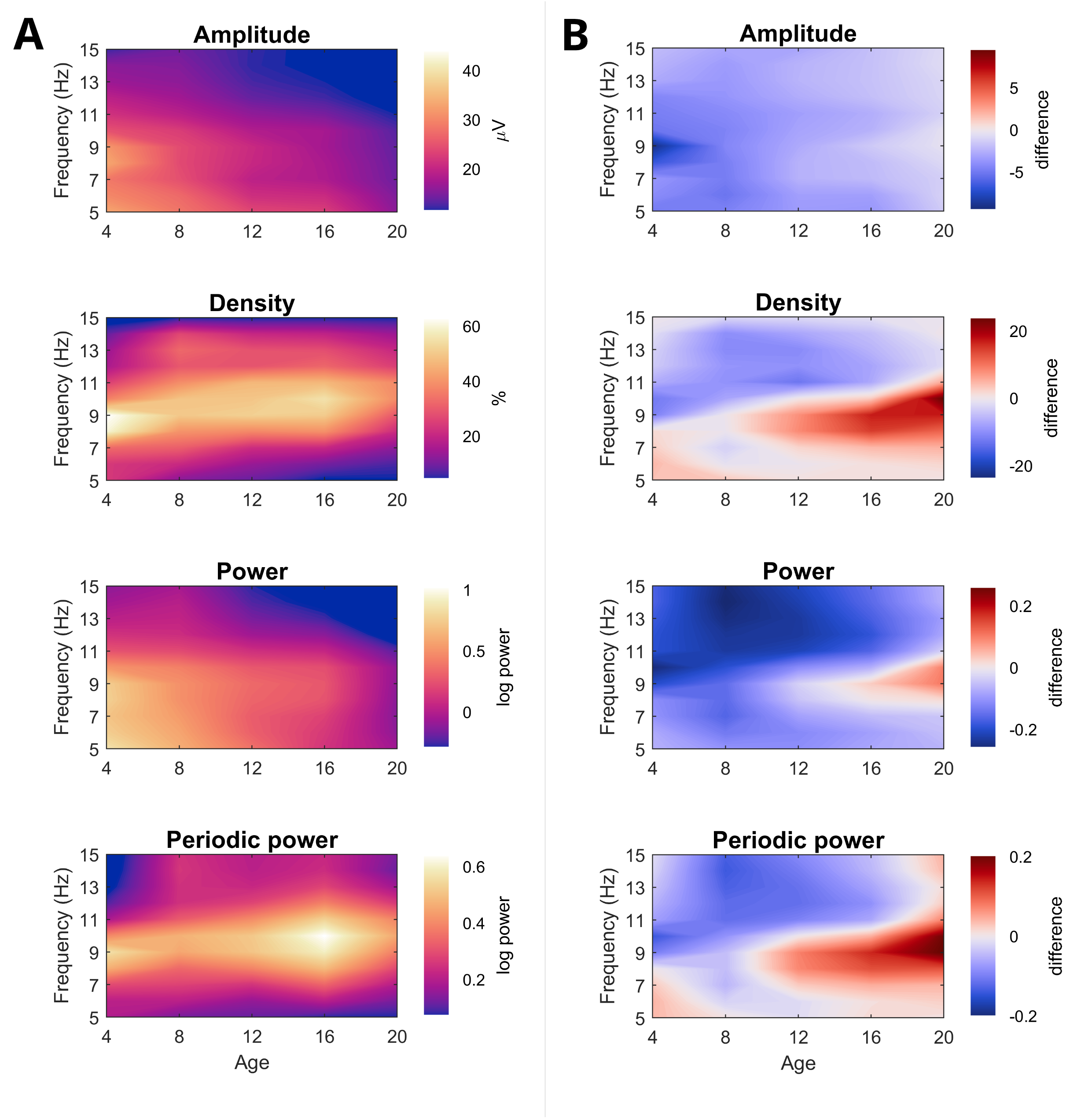

Supplement: Figure 6-1 — Average spectra across ages. From the oddball task, pooling controls and ADHD participants. Average spectra from the other tasks are provided in main Figure 2. A: Average values, such that lighter colors indicate greater magnitude for a given frequency and age. B: Difference values between morning and evening recordings, such that red indicate a greater magnitude in the morning. The measurement unit of each figure is the same as that of A. Download Figure 6-1, TIF file. [file eneuro-13-ENEURO.0384-25.2026-s005.tif]
