# Supplementary material for: The Interaction between Sleep and Development on Wake EEG Oscillations
Source: eNeuro. 2026 Apr 21;13(4):ENEURO.0384-25.2026. doi: 10.1523/ENEURO.0384-25.2026 (PMC13132015; doi:10.1523/ENEURO.0384-25.2026)
Supplement: Data 1 — MATLAB code used to preprocess, analyze and plot the wake EEG data used throughout the manuscript. Includes the primary repository children-wake/ as well as the required toolboxes: chART, fooof_mat, & Matcycle. Download Data 1, ZIP file. [file eneuro-13-ENEURO.0384-25.2026-s006.zip › Code/children-wake/functions/external/Mass_univariate_erp_toolbox/OLD/fileDifs.rtf]

*Files /Users/dgroppe/Desktop/Dropbox/PILE/Mass_Univariate_ERP_Toolbox/bin_info2EEG.m and /Users/dgroppe/GIT/Mass_Univariate_ERP_Toolbox/bin_info2EEG.m differOnly in /Users/dgroppe/GIT/Mass_Univariate_ERP_Toolbox: bin_mean.m <-is this file used? It is only in repo.*Files /Users/dgroppe/Desktop/Dropbox/PILE/Mass_Univariate_ERP_Toolbox/gui_erp.m and /Users/dgroppe/GIT/Mass_Univariate_ERP_Toolbox/gui_erp.m differ*Files /Users/dgroppe/Desktop/Dropbox/PILE/Mass_Univariate_ERP_Toolbox/sig_raster.m and /Users/dgroppe/GIT/Mass_Univariate_ERP_Toolbox/sig_raster.m differFiles /Users/dgroppe/Desktop/Dropbox/PILE/Mass_Univariate_ERP_Toolbox/topoplotMK.m and /Users/dgroppe/GIT/Mass_Univariate_ERP_Toolbox/topoplotMK.m differ
