# Supplementary material for: The Interaction between Sleep and Development on Wake EEG Oscillations
Source: eNeuro. 2026 Apr 21;13(4):ENEURO.0384-25.2026. doi: 10.1523/ENEURO.0384-25.2026 (PMC13132015; doi:10.1523/ENEURO.0384-25.2026)
Supplement: Data 1 — MATLAB code used to preprocess, analyze and plot the wake EEG data used throughout the manuscript. Includes the primary repository children-wake/ as well as the required toolboxes: chART, fooof_mat, & Matcycle. Download Data 1, ZIP file. [file eneuro-13-ENEURO.0384-25.2026-s006.zip › Code/children-wake/Manuscript_v11.docx]

The interaction between sleep and development on wake EEG oscillations

# Abstract

The amount of time previously spent awake or asleep strongly impacts the sleep electroencephalogram (EEG), especially slow waves during non-rapid-eye-movement (NREM) sleep. These effects on the sleep EEG meaningfully interact with age and to a lesser extent developmental disorders such as attention-deficit hyperactivity disorder (ADHD). We aimed to determine whether EEG oscillations during wakefulness were likewise affected by the interaction of sleep and development, using data collected from 163 participants 3-25 years old. We analyzed age- and sleep-dependent changes in two measures of oscillatory activity (amplitudes and density) and aperiodic activity (offsets and exponents). Finally, we compared wake EEG in children with ADHD (N=58) to neurotypical controls, with habitual good sleep quality required for inclusion. We found that oscillation amplitudes exhibited the same dynamics as sleep slow waves: decreasing with age, decreasing after sleep, and the overnight decrease decreasing with age. Strikingly, wake oscillation densities in the alpha band decreased overnight in children but increased overnight in adolescents and adults. Aperiodic measures were affected by both sleep and age albeit with minimal interaction. No wake measure showed significant effects of ADHD, suggesting that previously reported differences in patients may reflect uncontrolled variability in sleep quality rather than disorder-specific effects. While these results do not disentangle homeostatic from circadian effects, they underscore the need to control for sleep/wake history and measurement scheduling in all EEG experiments, especially when focusing on children and adolescents.

## Significance statement

Most studies measuring EEG during wakefulness do not take into consideration prior sleep/wake history. Here, we show that wake EEG measures significantly differ when measured before or after sleep, and these effects are strongly dependent on age. Differences between pediatric populations may in fact be due to prior sleep quality or circadian rhythms rather than hypothesized group differences.

# Introduction

The EEG is one of few tools available to study the human developing brain already from birth (Korotchikova et al., 2009), with the potential as a prognostic and diagnostic tool for both typical development and disease. Sleep EEG, and in particular slow wave activity (0.5-4 Hz) during NREM sleep, is especially sensitive to brain maturation (Campbell and Feinberg, 2009) and developmental disorders such as ADHD (Furrer et al., 2019). This is because slow wave activity reflects the overall synchronicity of the brain, which decreases with age following decreasing synaptic density across adolescence (Huttenlocher, 1979; Jenni and Carskadon, 2004; Campbell and Feinberg, 2009), and may be lower in ADHD due to reduced cortical thickness (Shaw et al., 2006).

In addition to development, sleep slow waves reflect the buildup and dissipation of homeostatic sleep pressure, increasing following wake and decreasing during sleep (Borbély, 1982). These changes in slow waves are hypothesized to reflect synaptic *plasticity*: synaptic strength increases with wake and daytime learning, and decreases during sleep (Tononi and Cirelli, 2003, 2014). Generally, plasticity decreases with brain maturation and this is reflected in decreases in the overnight *changes* in slow wave properties with age (Jaramillo et al., 2020). Thus, absolute slow wave activity reflects overall synaptic density, and changes in slow wave activity reflects plasticity. While these predictions have been repeatedly validated in sleep, their consequences on the wake EEG are less clear, due to limitations in prior analyses.

The EEG is made up of both *aperiodic* activity (the background signal) and *periodic* activity (oscillations) which combine together in the spectral domain as a characteristic 1/f curve with gaussian bumps (Figure 1A, Donoghue et al., 2020). Aperiodic activity is defined by its *offset* (reflecting the overall aperiodic power) and *exponent* (the steepness of the curve). Notably, aperiodic exponents become progressively steeper with increasing sleep depth (Lendner et al., 2020, 2023; Schneider et al., 2022), and become progressively shallower with increasing sleep duration, reflecting the dissipation of sleep pressure (Horváth et al., 2022; Bódizs et al., 2024). Brain maturation also strongly affects exponents, becoming shallower with age (Cellier et al., 2021; Hill et al., 2022; Tröndle et al., 2022), interacting with sleep depth (Favaro et al., 2023). Lastly, differences in aperiodic exponents and offsets have been found in children with ADHD compared to neurotypical controls (Robertson et al., 2019; Ostlund et al., 2021).

Periodic activity, like aperiodic activity, can be estimated from spectral power by subtracting the aperiodic activity from overall power, giving *periodic power* (Figure 1A). However, this ignores how oscillations can change in both *amplitude* and in *density* (Figure 1B). The amplitudes of oscillations reflect the synchronicity of the oscillating neuronal population, determined both by the number of neurons in phase with each other and the strength of their synaptic connections. Whether an oscillation occurs at all (i.e., density) will instead depend on the activity of “pacemaker” interneurons which entrain a population of neurons to the same rhythm (Perkel et al., 1964; Le Bon-Jego and Yuste, 2007), and this pattern of rhythmic firing will be in service of some underlying function that will come and go as needed. In short, amplitudes reflect synchronicity and densities reflect activity.

Because oscillation amplitudes reflect synchronicity, they should reflect the same information as slow waves measured during sleep. Sleep slow wave activity increases along a saturating exponential function relative to the prior duration of wakefulness, increasing rapidly after only a few hours awake and increasing less and less with further hours awake (Borbély, 1982; Dijk et al., 1987). In fact, wake oscillation amplitudes follow the same trajectory across 24 hours awake (Snipes et al., 2023). Instead, alpha oscillation densities (8-12 Hz) decreased, supporting the specificity of the effect to amplitudes and masking the effect when measuring spectral power.

Given these prior results, we hypothesized that wake oscillation amplitudes specifically should behave like sleep slow waves also across development: absolute amplitudes should decrease with age, and overnight changes in amplitude should decrease with age. Furthermore, given that children with ADHD have lower sleep slow wave activity, they should likewise have lower wake amplitudes.

To answer these questions, we analyzed data collected from previous studies at the University Children's Hospital of Zurich, with high-density wake EEG recordings measured the evening before and morning after a night of sleep. The final dataset included 105 neurotypical participants and 58 patients with ADHD.


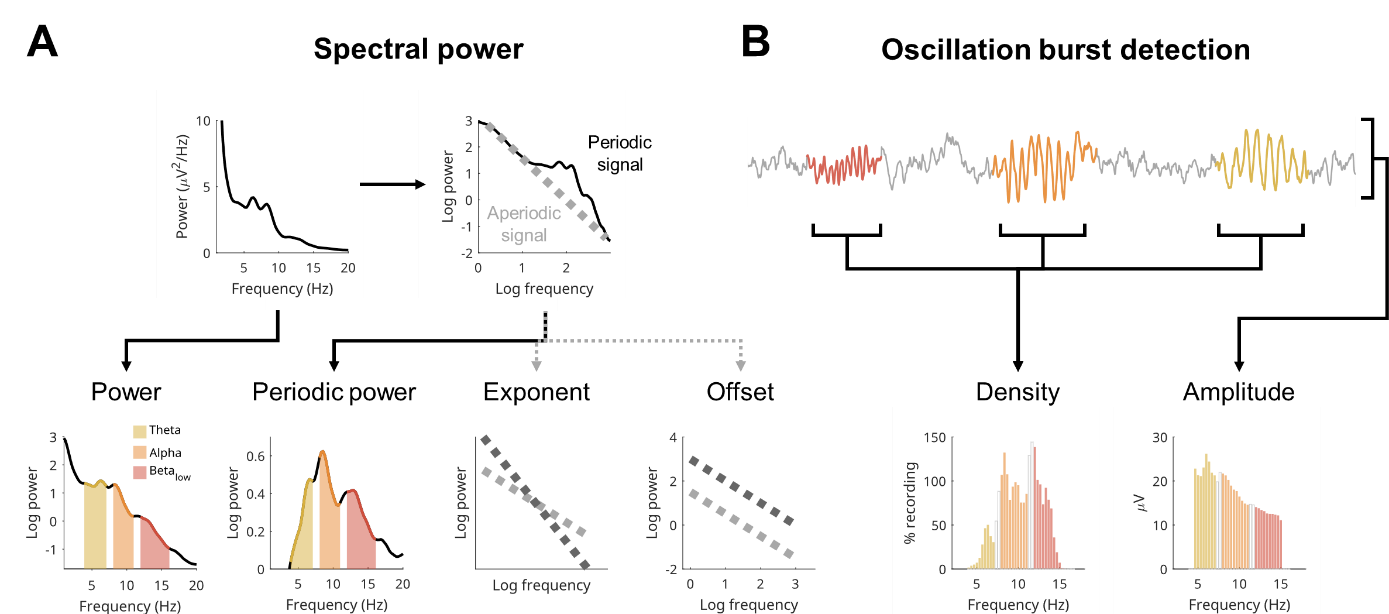


**Figure 1: Wake EEG measures. A**: Measures based on spectral power. Given the nature of power, it is traditionally analyzed log-transformed to have more normally distributed values. These are then aggregated into bands. Here, we focus on classical wake EEG bands: theta (4-7 Hz, yellow), alpha (8-11 Hz, orange) and low beta (12-16 Hz, red), with gaps between bands to avoid overlapping information. The example comes from a 15-year-old male participant, used for the entire figure. The EEG signal is composed of aperiodic “background activity” (gray signal in B) and oscillatory activity (colored signal in B). When plotting the power spectrum on a log-log scale, a line can be fitted to the aperiodic component of the signal, which can be subtracted from the whole spectrum, leaving behind only periodic power. The aperiodic line can then be quantified by its offset (where it intersects 0 on the log-log scale, i.e. the log power at 1 Hz), and its exponent (how tilted it is). Thus, the power spectrum provides four measures: log-transformed power, periodic power, exponent, and offset of the aperiodic signal. **B**: Cycle-by-cycle analysis is used to detect bursts of oscillations by identifying sections of the EEG signal that show primarily periodic activity (colored), relative to the aperiodic background activity (gray). Once bursts are detected, there are two main parameters to quantify them: density (how much of the signal in time contains an oscillation) and amplitude (average peak-to-peak voltage of each oscillation). Densities are expressed in percentage, and when pooling bursts detected in all channels, they can easily exceed 100%, as the same burst will appear in multiple channels and multiple bursts can co-occur. Amplitudes are in microvolts. The EEG trace was stitched together for illustrative purposes. N.B. Beta periodic power is lower than alpha periodic power (in panel A), but their densities (in B) are roughly the same; this is because periodic power is also influenced by the lower amplitudes of low beta compared to alpha. The densities of other participants are provided in Extended data Figure 1-1.

# Methods

## Datasets

The data for this manuscript was assembled from previous studies conducted between 2008 and 2021. The participant demographics of each dataset are in Table 1. In total, we included 163 participants between the ages 3.5 and 24.7, 38% female, 7% left-handed. Of these, 36% were diagnosed with ADHD at the department for Child and Adolescent Psychiatry at the University of Zurich, the outpatient clinic of the Child Development Center, and at private children’s clinics in Zurich Oerlikon. Patients were not excluded based on medication status, and therefore were a mixture of medicated, previously medicated, and unmedicated (see Table 2, and (Ringli et al., 2013; Furrer et al., 2019)). Otherwise, all participants were screened by telephone such that they all were completely healthy, took no (other) medication, had no (other) comorbidities, and were good sleepers. All participants were recruited from canton Zurich, Switzerland, and recorded at the University Children's Hospital of Zurich, except for the children from 3.5 to 8 (Dataset2009, N=11), who were recruited in Providence, RI, USA, and recorded at home. Sleep time was determined by their individual preferred sleep and wakeup time, which they had to maintain the week prior to each measurement. Wake measurements were done just before going to sleep, and ~30 minutes after waking up. 115 participants had 2 sessions, spaced at least 1 week apart, both included in these analyses. Depending on the dataset, different paradigms were used involving different wake tasks (described below) and there could be 1-4 tasks at each time point (see Table 1). Every set of tasks for each dataset was repeated in the evening and in the morning. In total, 1243 recordings were included in these analyses lasting on average 4.2 min (task time + buffer − artefacts), with a standard deviation of 2.5 min. The processed data for all participants is provided as extended data (ExtendedData_1, ExtendedData_3).

Informed consent was obtained from all adult participants, and from the legal guardians of all children below 14, as well as from adolescent participants 14-18. All studies were approved by the local ethics committees and performed according to the declaration of Helsinki.

|  | N  # | Female  % | Left-handed  % | ADHD  % | Age range  (years) | Mean age  (years) | Paradigm | Sessions  # | Recordings  # |
| --- | --- | --- | --- | --- | --- | --- | --- | --- | --- |
| Dataset2008 | 38 | 32 | 0 | 0 | 8.7-23.4 | 14.1 (3.8) | Adaptation | 2 | 7.3/8 |
| Dataset2009 | 11 | 73 | 0 | 0 | 3.5-8.0 | 5.6 (1.5) | Oddball | 1 | 1.8/2 |
| Dataset2010 | 28 | 21 | 14 | 100 | 9.7-16.3 | 12.7 (1.9) | Adaptation | 1 | 2.8/4 |
| Dataset2016 | 18 | 44 | 0 | 0 | 18.4-24.7 | 21.6 (2.1) | Oddball | 2 | 3.9/4 |
| Dataset2017 | 42 | 43 | 17 | 36 | 8.1-17.6 | 12.2 (2.7) | Attention | 2 | 12.7/16 |
| Dataset2019 | 26 | 38 | 0 | 58 | 8.8-16.8 | 11.4 (2.0) | Attention | 2 | 10.1/12 |
| All | 163 | 38 | 7 | 36 | 3.5-24.7 | 13.2 (4.4) |  |  |  |

**Table 1: All participants’ demographics, split by dataset.** The year for each dataset indicates the beginning of data collection. *N* indicates the number of participants. After the mean age of each dataset, the standard deviation is provided in parentheses. *Paradigm* indicates which set of wake tasks were recorded. *Sessions* indicate the number of sessions expected for each dataset, although in practice due to dropouts, some participants only completed 1. *Recordings* indicate the average number of recordings per participant compared to the total number of recordings expected by the experimental paradigm. Recordings were missing either because they contained too many artefacts or were omitted entirely during data collection.

|  | N  # | Female  % | Left-handed  % | Age range  (years) | Mean age  (years) | Oddball  % |
| --- | --- | --- | --- | --- | --- | --- |
| Medicated in the past (1) | 6 | 17 | 0 | 9.7-14.8 | 12.5 (2.2) | 33 |
| Unmedicated (2) | 14 | 21 | 14 | 9.5-15.3 | 11.3 (1.6) | 64 |
| Medication the day before (3) | 22 | 23 | 14 | 8.7-16.3 | 12.3 (2.2) | 45 |
| Medication the day of (4) | 13 | 15 | 0 | 9.0-16.1 | 12.3 (2.0) | 54 |
| All patients | 55* | 20 | 9 | 8.7-16.3 | 12.1 (2.0) | 51 |
| Controls (5) | 105 | 47 | 6 | 3.5-24.7 | 13.8 (5.2) | 58 |

**Table 2: ADHD demographics, split by patient status.** The indices in parentheses provides the subgroup categories in Extended Data 1. For each patient group, the *Oddball* column indicates how many came from a dataset performing the oddball task (rather than the attention tasks). *For 3 patients, medication status was missing, therefore the true total is 58.

### Oddball & motor adaptation paradigms

95 participants (Dataset2008, Dataset2009, Dataset2010, Dataset2016) performed an auditory oddball task during their wake EEG. The task lasted 4 minutes and was performed in the evening just before going to bed and in the morning ~30 minutes after waking up. The task involved 300 tones at ~80 dB, with an interstimulus interval of 0.8 s. A random 10% of stimuli were targets to which the participant had to push a button in response. For the youngest children (Dataset2009), the 4-minute task was split into 2 segments, and for the adults (Dataset2016) the task was extended to 6 minutes.

66 of these participants (Dataset2008, Dataset2010) also performed a half-hour visuomotor adaptation task (Ghilardi et al., 2000) followed by a second oddball. One dataset (Dataset2008) also included a second session with a control visuomotor task (no adaptation), counterbalanced with the motor adaptation task. The motor tasks were not included in this analysis, because they further differed from evening to morning. For more details on the adaptation task see Wilhelm et al. (2014). The youngest (Dataset2009) and oldest (Dataset2016) participants only conducted one oddball and no motor task. Dataset2016 also had two sessions, one night with phase-targeted auditory stimulation during NREM sleep and the other sham. The experimental conditions did not affect the wake EEG (data not shown).

The sleep data from these participants has been published (Kurth et al., 2010; Buchmann et al., 2011; Ringli et al., 2013; Wilhelm et al., 2014; Volk et al., 2018; Furrer et al., 2019, 2020; Jaramillo et al., 2020), as has a subset of the wake EEG data (Fattinger et al., 2017).

### Attention paradigm

68 participants (Dataset2017, Dataset2019) performed three tasks with a focus on attention. These were studies investigating the relationship between slow waves, behavior, and MR spectroscopy (Volk et al., 2019; Jaramillo et al., 2020). This included two sessions to compare the effects of phase targeted auditory stimulation on slow waves in sleep (sham and stimulation; data currently unpublished). The wake tasks were part of the Test Battery for Attentional Performance (TAP) (Zimmermann and Fimm, 2012), which included 2 minutes of a visual Go/No-Go task (respond to 1 stimulus, withhold response to another), 5.5 minutes of a visual and acoustic Alertness task, and then 2 1.5-minute Fixation recordings. For one dataset (Dataset2019) the Go/No-Go task was adapted using cartoon images (extended to 12 minutes), but only 1 Fixation recording was measured (lasting 2 minutes).

## EEG recordings and preprocessing

All datasets were measured using 128 channel EGI Geodesic Sensor nets and EGI amplifiers (Electrical Geodesics Inc., Eugene, OR, USA). Wake recordings were done with Cz reference, 1000 Hz sampling rate, and impedances kept below 50 kOhm. All analyses were performed in MATLAB 2023b, with the EEGLAB toolbox v2023.1 (Delorme and Makeig, 2004), the FOOOF/specparam toolbox v1.1.0 (Donoghue et al., 2020), and custom scripts.

EEG data was first mean-centered, then lowpass filtered at 40 Hz (EEGLAB’s *pop_eegfiltnew* function) and notch-filtered at either 50 or 60 Hz (Dataset2009) along with subsequent harmonics. The data was downsampled to 250 Hz, then highpass-filtered over 0.5 Hz (Kaiser filter, stopband = 0.25 Hz, stopband attenuation = 60, passband ripple = 0.05).

Artifacts were removed with a fully automated procedure. Movement and other large artifacts were detected in data filtered between 1 and 40 Hz, in 3 s segments. A segment was labeled a “major artifact” if it exceeded 500 μV, or a “minor artifact” if the correlation with neighboring channels was below .3. Major artifacts were always removed, either by removing all data in all channels during those 3 s, or removing the entire channel with such an artifact, depending on which (channel or segment) removed the least amount of clean data. Minor artifacts were removed in a similar way, removing iteratively either the channel with the most artifactual segments, or the segments with the most artifactual channels, until all channels and all segments had at most 30% of the data containing a minor artifact. Flat channels were removed using EEGLAB’s *clean_artifacts* function. The missing Cz channel was added (as a vector of zeros), then the data was average referenced. Physiological artifacts (blinks, eye movements, muscle tone, heartbeat) were removed with independent component analysis (ICA), with components calculated separately as described in the next section. After these were removed, a second pass was conducted using EEGLAB’s *clean_windows* function (MaxBadChannels = .3, PowerTolerances = [-inf, 12]), then bad segments/channels still containing amplitudes over 140 μV were removed, and finally EEGLAB’s *clean_channels_nolocs* was applied (MinCorrelation = .5, IgnoredQuantile = .1, MaxBrokenTime = .5). Recordings for which more than 25 channels were removed, or which had less than 1 minute of data, were excluded from analysis. In a last step, EEG channels were interpolated, for a total of 123 channels, excluding the external electrodes (49 56, 107, 113) and the face electrodes (126, 127).

### Automatic detection and removal of physiological artefacts using ICA

For ICA, a separate copy of the EEG data was preprocessed as previously described, however the data was filtered between 2.5 and 100 Hz, and downsampled to 500 Hz. Automatically detected bad channels and bad time windows were removed, an empty Cz channel added, and then the data was re-referenced to the average of all channels. EEGLAB’s *runica* function was run with principal component analysis (PCA) rank reduction. Then, components were automatically classified with EEGLAB’s *iclabel*, as either brain, muscle, eye, heart, line, channel noise, or other. This function provides a probability score for each label from 0 to 1, so the label with the largest score for each component was taken. Components classified as muscle, eye, or heart were removed. Of the remaining noise classifications (line, channel, other), due to poor classification accuracy, an additional step was implemented. Spectral power was calculated for each component (*pwelch*, 4 s Hanning windows, 50% overlap), then smoothed over 5 Hz (*lowess*) to facilitate model fitting. Unlike for the main analyses, the periodic signal was not of interest, therefore stronger smoothing was possible. The *specparam* algorithm (Donoghue et al., 2020; Ostlund et al., 2022) was applied to the power spectrum between 8 and 30 Hz to extract aperiodic exponents. Components for which the spectral exponent was shallower than 0.5 (so almost flat or even tilted positive), were considered noise and therefore excluded, as they reflected either muscle activity or other non-physiological signals. Using the manually labeled components in an independent adult dataset (Snipes et al., 2022), we confirmed that this procedure was sufficiently comparable to human detection of artifactual components. We further confirmed that the outcome matched human component classification in a small subset of the children’s data. However, considering the trend towards 0.5 exponents observed in Figure 3, for future datasets with older participants we would recommend a lower threshold.

For the Dataset2009 cohort of <8-year-olds, given how little data there was and how many more movement artifacts, we chose to apply to same manual artifact rejection as in Snipes et al. (2022) to preserve as much data as possible.

## Burst detection

Bursts of oscillations were detected using cycle-by-cycle analysis (Cole and Voytek, 2019) implemented in MATLAB (Snipes et al., 2023). Bursts were detected with the same thresholds as in Snipes et al. (2024). Briefly, EEG was narrow-band-pass filtered in overlapping ranges (2-6 Hz, 4-8 Hz…), from which zero-crossings were detected. Then, in the broadband filtered data (0.5-40 Hz), peaks were identified between the zero-crossings, and a cycle was considered an oscillation from positive-to-positive peak. A minimum number of consecutive cycles must meet a set of criteria (monotonicity, period consistency, amplitude consistency, shape consistency, etc.) for this to be considered a burst. Importantly, amplitude itself is never used as a threshold, as this would create a greater dependency between amplitude and density (such that a decrease in an amplitude threshold would result in an automatic increase in density).

Three sets of criteria were used. The first aimed to detect bursts relying on many low-threshold criteria (frequency in range of narrowband filter; PeriodConsistency = .5; AmplitudeConsistency = .4; FlankConsistency = .5; ShapeConsistency = .2; MonotonictyInTime = .4; MonotonicityInAmplitude = .4; ReversalRatio = .6; MinCycles = 4). The second had fewer criteria with intermediate thresholds but a higher minimum number of cycles (PeriodConsistency = .6; AmplitudeConsistency = .6; MonotonicityInAmplitde = .6; FlankConsistency = .6; MinCycles = 5). The third set had fewer criteria but stricter monotonicity thresholds (frequency in range of narrowband filter; PeriodConsistency = .7; FlankConsistency = .3; MontonocityInAmplitude = .9; MinCycles = 3). These criteria were chosen a-priori based on manual tuning of the burst detection on an independent dataset of wake EEG in adults during sleep deprivation.

After bursts were detected in each channel separately, they were grouped into clusters when they occurred simultaneously in multiple channels with roughly the same frequency. The frequency of bursts was calculated as the inverse of the average distance between negative peaks (1/period). Bursts for which the shorter one overlapped at least 50% of the longer one, and were within 1 Hz of each other, were considered part of the same burst cluster. Bursts identified separately in each channel were used for all the topographies, otherwise burst clusters were used to reduce the effect of burst globality (spread across the scalp) on measures of density.

### Oscillation measures

*Oscillation amplitudes* were calculated as the average negative-to-positive peak voltage difference for all cycles involved in all bursts, with units in microvolts (μV). *Oscillation densities* were calculated as the percentage of the recording occupied by bursts (sum of all the bursts’ durations divided by the duration of the recording). This accounts for both the duration and overall quantity of bursts in the recording. The average duration and number of bursts can be affected by the background aperiodic signal which can break up sustained oscillations into smaller bursts (Tal et al., 2020), therefore burst density was preferable. When calculating across multiple channels (e.g. Figure 3), oscillation density could easily exceed 100%, as burst clusters in different frequency ranges often co-occur. When combining densities across multiple frequency bands, bursts were pooled rather than averaged (sum of the durations of all the bursts of any frequency, divided by the duration of the recording).

### The choice of frequency bands

Only bursts between 2 and 16 Hz were detected. Below 4 Hz very few bursts could be identified, therefore only bursts above 4 Hz were included in the analysis. Bursts over 16 Hz could be detected, but with higher false-positive rates, as determined by visual inspection. The choice of cutoff at 16 Hz was done arbitrarily a-priori to capture alpha (8-12 Hz) with generous padding. The division of bands for Figure 6 and Figure 7 was done using conventional bands (Pernet et al., 2020) with 1 Hz gaps to reduce overlapping information due to the drift in peak frequencies across individuals and ages: theta (4-7 Hz), alpha (8-11 Hz) and low beta (12-16 Hz). The inclusion of low beta was done a-posteriori based on results observed in B.

Many researchers advocate for the use of an individual alpha frequency (IAF) to define frequency ranges, especially when analyzing data across development (Klimesch, 1999; Bazanova and Vernon, 2014; Ostlund et al., 2022; Tröndle et al., 2022). This is done by selecting the peak alpha frequency separately for each individual and defining the band around this peak. The shift in IAF with age makes a strong case for such an approach (Freschl et al., 2022). The main problem with using IAF is that it assumes the largest oscillation will be functionally the same for all participants. Given that participants displayed large heterogeneity in the number and amplitude of frequency peaks within the 2-16 Hz range (extended data Figure 1-1), and the vast majority were old enough that the peak alpha frequency was larger than 8 Hz (Freschl et al., 2022), we preferred to use fixed bands with gaps. The completely distinct topographies across the three bands for all age groups support this decision for this dataset (Figure 6, Figure 7).

## Spectral power analysis

Spectral power was calculated using MATLAB’s *pwelch* function, with 4 s Hanning windows and 50% overlap. When average power across channels was calculated, edge channels were excluded (total count: 98). To dissociate periodic and aperiodic spectral power, we used the MATLAB extension of specparam (formerly known as FOOOF (Donoghue et al., 2020)). Spectra were smoothed over 2 Hz, and the aperiodic signal was fitted between 2 and 35 Hz (frequencies sufficiently separated from the 0.5-40 Hz filter range of the preprocessed data). Otherwise, the default settings were used (peak width: [0.5 12], max number of peaks: inf; minimum peak height: 0; peak threshold: 2; aperiodic mode: fixed).

### Spectral and aperiodic measures

*Power* was calculated by averaging the log-transformed power values between 4 and 16 Hz. Aperiodic *offsets* were provided by specparam as the log power value at 1 Hz of the aperiodic signal, and *exponents* as the x value in the $1/f^{x}$ model that describes the steepness of the aperiodic signal. The values are such that positive exponents refer to a downward descending aperiodic signal, and the larger the value the steeper the descent. *Periodic power* was calculated as the log-transformed power from which the aperiodic signal was subtracted. Fitting model parameters *r-squared* and *mean absolute error* (MAE) were similarly analyzed to evaluate whether the model fit could account for the results (Ostlund et al., 2022), with the results provided in extended data Figure 2-1. Overall, the specparam model fit was very good, with average MAE of 0.028 (interquartile range: 0.019, 0.035) and r-squared values of 0.998 (0.997, 0.999).

## Statistics

Statistics were performed using the MATLAB Statistics and Machine Learning Toolbox. For all analyses, statistical significance was determined for p-values < .05. Given the heterogeneous datasets pooled together for this analysis, we chose to conduct linear mixed effects models (MATLAB function *fitlme*) to model the relationship between age, sleep, ADHD, and EEG measures. Each model used is provided in proximity to the corresponding results section, for clarity.

For each fixed factor of the model, β estimates, t-values, p-values, and degrees of freedom (df) are reported in the text. β estimates of continuous variables (e.g., age) indicate by how much the EEG measure (e.g. density) changes for each unit of the continuous variable (e.g., 1 year) when all other variables are 0. Similarly, the β estimates of categorical variables (e.g. group) indicate how much the EEG measure changes from that category (e.g., ADHD) to the baseline category (e.g., controls), for all other factors set to 0. Lastly, t-values allow a comparison of the magnitude of the effect of each factor when comparing models with different measuring units.

To determine the topography of the effects, we ran linear mixed effects models for each channel separately, correcting for multiple comparisons using false discovery rates (FDR; (Benjamini and Yekutieli, 2001)). We plotted the FDR corrected p-values and corresponding β estimates to identify regions showing significant overnight changes in each outcome variable.

We ran models with fixed factors Task, Time, Age, Group, and Sex, the interaction between Time and Age, and nested mixed factors Participant and Session. *Task* compared the levels *Oddball* vs. *go/no-go*, *alertness*, and *fixation*. *Time* compared the time of recording; evening vs. morning for wake, and first vs. last hour of NREM sleep. *Group* compared neurotypical participants vs. those with ADHD. *Sex* compared females vs. males. Depending on the analysis and subset of the data, different factors were included or not, and so the exact model is specified before each analysis and in each figure caption.

As a simpler analysis and sanity check, we conducted Pearson’s correlations between age and each measure, across all recordings (Figure 3). In each figure, r values are provided as effect sizes. We further ran Pearson’s correlations between measures to evaluate the relative importance of the four main measures with spectral power and periodic power (Section 3.1.1). Both sets of correlations used FDR correction for multiple comparisons.

To determine whether differences in r values were statistically significant, we ran dfggfor dependent correlations sharing one variable (Steiger, 1980; Lee and Preacher, 2013).

All data used for the channel-averaged statistical models are provided as Extended Data 1 and the statistical models as Extended Data 2.

# Results

## Effects of age, sleep, sex, and ADHD on wake EEG measures

The following linear mixed effects model was applied to each wake EEG measure, averaged across channels: $Measure \sim Task + Time*Age + Group + Sex + (1|Participant) + (1|Participant:Session)$. The full outputs of the model are provided in Extended Data 2. Average spectra are provided in Figure 2 and the relationship between age and each measure is provided in Figure 3.

Oscillation amplitudes significantly decreased with age (β = -0.783, t = -11.33, p < .001, df = 1234), significantly decreased the morning following sleep (β = -3.977, t = -15.90, p < .001, df = 1234), with a significant positive interaction (β = 0.143, t = 7.74, p < .001, df = 1234), such that amplitudes decreased less overnight with increasing age (Figure 3, bottom row). Amplitudes were significantly lower in males than females (β = -1.476, t = -2.38, p = .017, df = 1234), and were not significantly different in participants with ADHD (β = -0.574, t = -0.89, p = .373, df = 1234). As can be seen in Figure 3, the relationship between age and amplitudes was quite robust, both as absolute values (r_eve_ = -.66, r_mor_ = -.63) and overnight changes (r = .56). Overall, amplitudes changed in the expected directions for both development and sleep pressure, except for the lack of an effect of ADHD.

Oscillation densities significantly decreased with age (β = -3.540, t = -2.11, p = .035, df = 1234), and the morning after sleep (β = -77.506, t = -10.39, p < .001, df = 1234), with a significant positive interaction between age and time of recording (β = 4.878, t = 8.85, p < .001, df = 1234). Unlike amplitudes, the correlation between age and density was weak (r_eve_ = -.28, r_mor_ = .01). Instead, the correlation between age and overnight change in density was quite strong (r = .57), such that oscillation densities decreased overnight in children under 15 and increased in young adults (Figure 3). There was no effect of ADHD (eta = -0.154, t = -0.01, p = .992, df = 1234) or sex (β = -16.446, t = -1.10, p = .271, df = 1234). Overall, oscillation densities behaved independently from amplitudes, especially in the direction of overnight changes in adolescents and adults.

Aperiodic exponents became significantly shallower with age (β = -0.027, t = -9.23, p < .001, df = 1234) but significantly steeper overnight (β = 0.099, t = 5.51, p < .001, df = 1234), with a trending negative interaction between time of recording and age (β = -0.003, t = -1.91, p = .056, df = 1234). The increased overnight steepness was driven primarily by a decrease in higher frequency power (Figure 2), whereas decreasing steepness with age is driven by decreases in low frequency power (Favaro et al., 2023). The correlations between exponents and age were as robust as for oscillation amplitudes (r_eve_ = -.60, r_mor_ = -.66), but the correlation with overnight change was weak (r = -.15, not significant). There was no significant effect of ADHD (β = 0.028, t = 1.07, p = .284, df = 1234) or sex (β = 0.041, t = 1.63, p = .102, df = 1234). This means that aperiodic exponents also change independently from oscillation amplitudes, and do not reflect the direction of changes expected for sleep pressure.


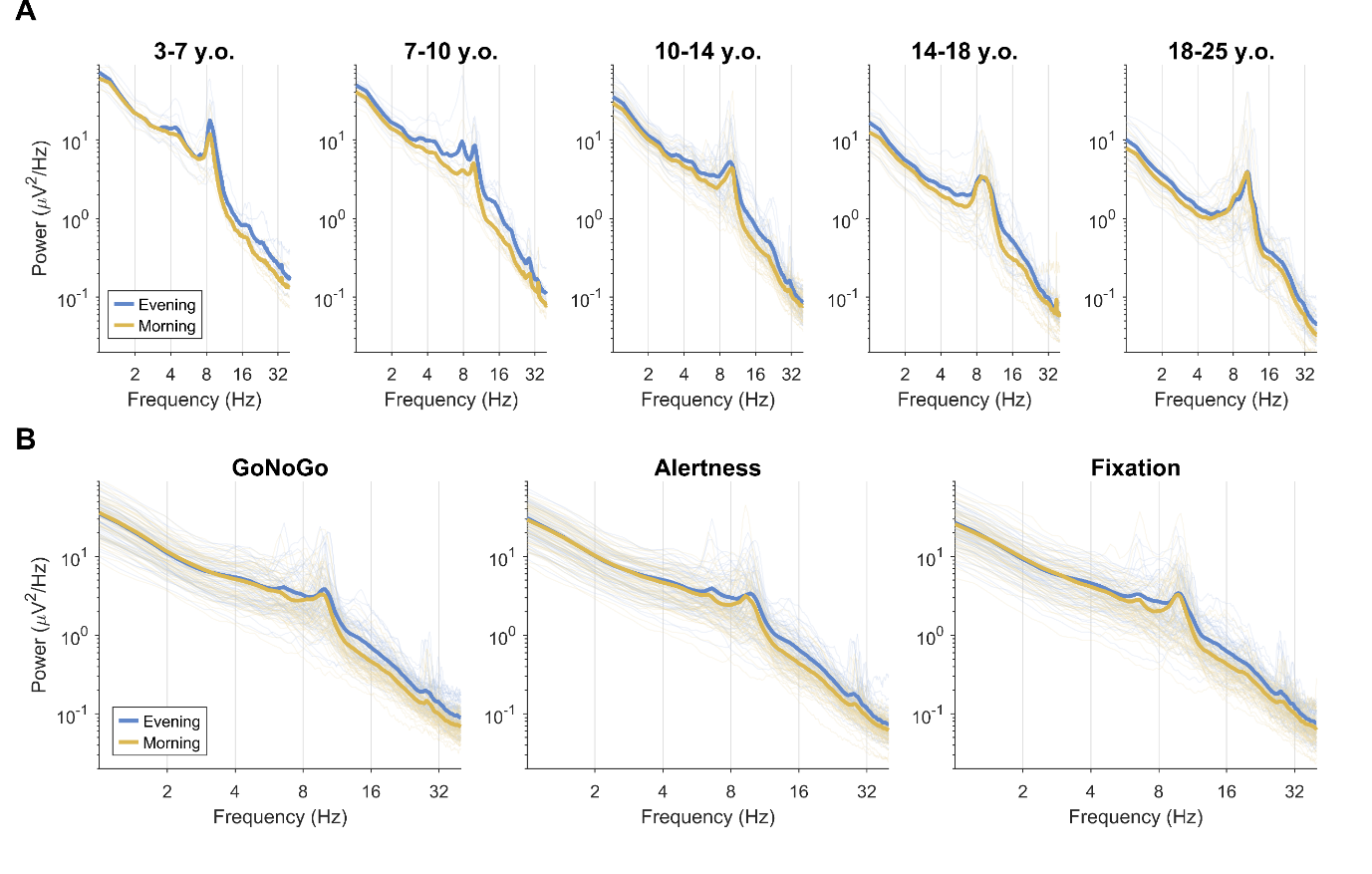


**Figure 2:** Log-log power spectra, averaged across channels. Thick lines indicate group averages, thin lines indicate individuals (averaged across multiple recordings and/or channels), separately in the evening (blue) and morning (yellow). ADHD and controls are pooled. X- and y-axes are plotted on logarithmic scales. **A:** Auditory oddball tasks, split by age group, from datasets 2008, 2009, 2010, 2016. **B:** Go/no-go, alertness, and fixation recordings from datasets 2017 and 2019. The peak of activity ~30 Hz reflects iota oscillations (Snipes, 2025), shown here to be largely task-independent.

Aperiodic offsets also significantly decreased with age (β = -0.067, t = -16.59, p < .001, df = 1234), but with no significant effect of time (β = 0.029, t = 1.65, p = .100, df = 1234), and a significant negative interaction (β = -0.004, t = -3.11, p = .002, df = 1234), such that offsets decreased more overnight with age. The correlations between age and offsets were the strongest of all measures (r_eve_ = -.81, r_mor_ = -.83), however, the correlation between age and overnight change in offset was negligible (r = -.05). Again, there was no effect of ADHD (β = 0.021, t = 0.55, p = .579, df = 1234) or sex (β = -0.003, t = -0.07, p = .944, df = 1234). Overall, offsets correlated with age in the same direction as amplitudes, changed overnight in the same direction, but the overnight change was not larger in children compared with adults, counter to what would be expected for sleep pressure.

Curiously, fitting parameters of the specparam algorithm showed both significant age and time of recording effects, with the model fit improving in the morning and worsening with age (extended data Figure 2-1). Therefore, differences in fit quality may partially contribute to the effects observed for aperiodic measures. However, the changes in model fitting go in the opposite direction one would expect of data quality (i.e. worse in the morning and better in adults). This suggests that some other aspect of the EEG signal that changes with age and time can affect the fit quality of the specparam algorithm.


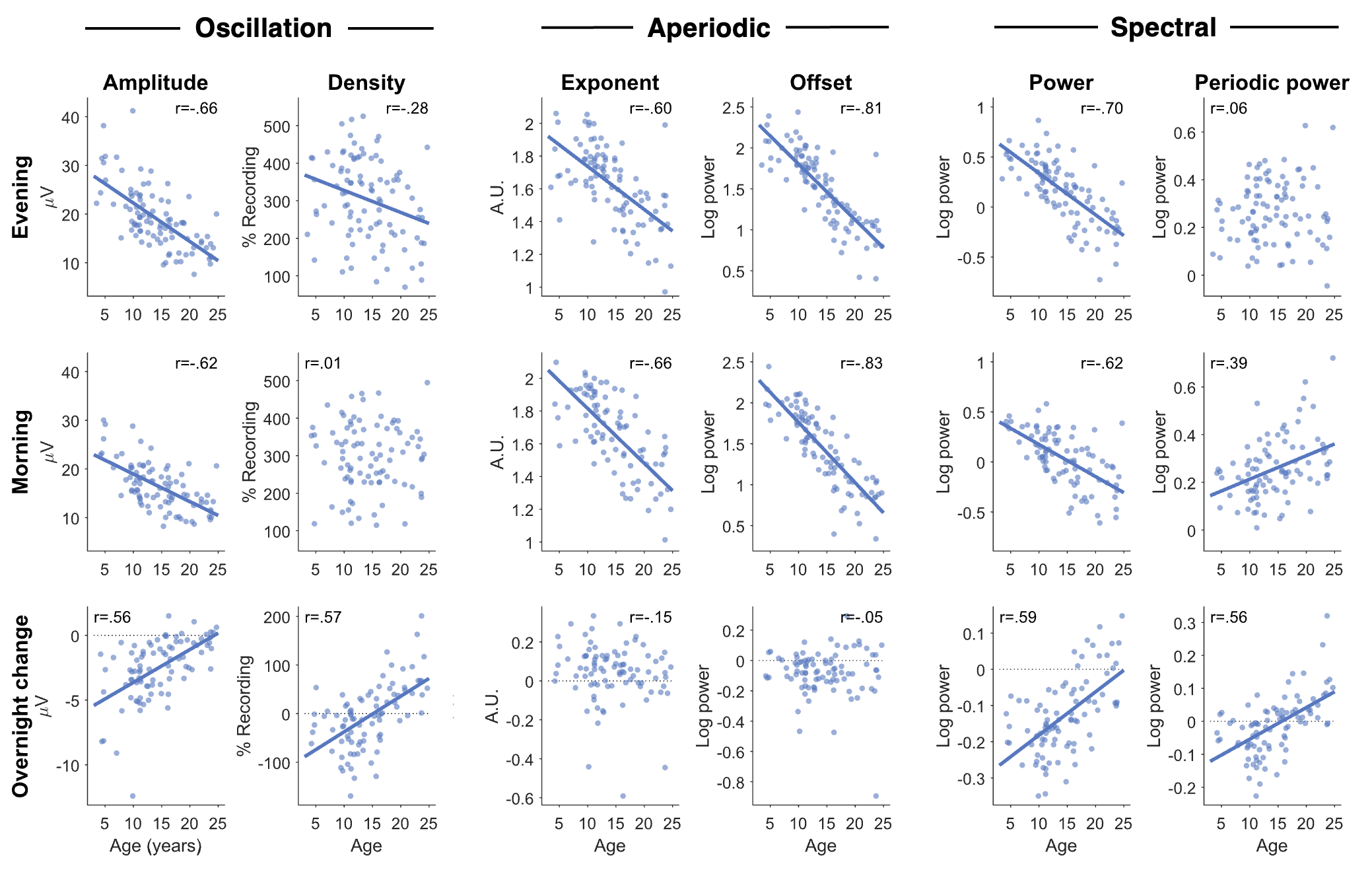


**Figure 3: Wake EEG measures correlated with age.** Only auditory oddball recordings were included, pooling both neurotypical and ADHD participants. Each dot represents the value for a single participant. For participants with multiple sessions, values across sessions were first averaged. Pearson’s correlations were done for each figure, with rho values provided in the corner. If the p-value was less than .05, a correlation line was drawn (FDR correction for multiple comparisons). Amplitudes and densities of oscillations were obtained from burst clusters, pooling all frequencies (4-16 Hz). Exponent values are such that larger values indicate a steeper aperiodic signal; units are “arbitrary” (a.u.), in that exponents are scale-free measures. Power spectra were calculated for each channel, then averaged across all channels, excluding the outermost ring of channels. Power and periodic power were then calculated by averaging values from 4 to 16 Hz. The same figure for the specparam fitting estimates is provided as extended data Figure 2-1. The correlations between each measure with the other is provided in extended data Figure 2-2.

In summary, of the four EEG measures, only amplitudes followed the same trajectories expected for both development and sleep pressure. The absolute values of all four measures had a negative correlation with age, and differed primarily in the overnight response and the relationship between age and overnight response. Oscillation densities in particular showed a strong effect of age on overnight changes, reversing direction between childhood and adolescence. No measure showed any relationship with ADHD, and only amplitudes were affected by sex. Therefore, in later analyses we did not include these factors, and pooled patients and controls for greater statistical power.

### Comparison of oscillation and aperiodic measures to spectral measures

To quantify the extent to which spectral measures were influenced by oscillation and aperiodic measures, we directly correlated each measure to the other (extended data Figure 2-2), and then controlled for time of the recording, task, and age using linear mixed effects models: $Measure1 \sim Measure2 + Time*Age + Task + \left( 1 | \mathrm{Participant} \right)+ \left( 1 | Participant:Session \right)$ (extended data Table 2-1).

Using FDR to correct for multiple comparisons, all measures significantly correlated with each other, except periodic power which did not correlate with either aperiodic exponents or offsets. Power was most related to amplitudes, also in the mixed effects model (correlation r = .90, mixed effects t-value = 39.3). Power was also highly related to offsets (r = .78, t = 17.5), but less so with densities (r = .61, t = 28.2) and exponents (r = .48, t = 4.7).The difference in r values between the amplitude-power correlation and the others was statistically significant (z > 12.43, p < .001). Periodic power was highly correlated with densities (r = .85, t = 46.9) and somewhat with amplitudes (r = .43, t = 20.6). Again, the difference in r values was statistically significant (z = 23.96, p < .001).

## Topography of wake EEG measures by age

Figure 4 provides the average topographical maps of each measure for five age bins, averaging (or pooling for densities) all frequencies from 4-16 Hz, from the oddball task. Amplitudes, densities, exponents, and offsets all showed unique topographies from each other. Across ages, for each measure there were primarily changes in magnitude more so than major regional differences. However, oscillation amplitudes in the youngest cohort began as a single midline occipital spot, which spread bilaterally in the 7–10-year-olds. Prominent central bilateral peaks also appeared in the 7–10-year-olds. Oscillation densities similarly started as a single midline occipital spot, but these became more lateral-parietal in the 14–18-year-olds. Like amplitudes, two small bilateral central peaks emerged in the densities of 7–10-year-olds, which merged with the primary occipital-parietal cluster in the 14–18-year-olds. Furthermore, a frontal peak gradually emerged with age. Exponents were steepest in midline channels, whereas offsets showed both a frontal midline and occipital peak. As with the correlations between measures, power topographies most resemble amplitudes, and periodic power resembles densities.


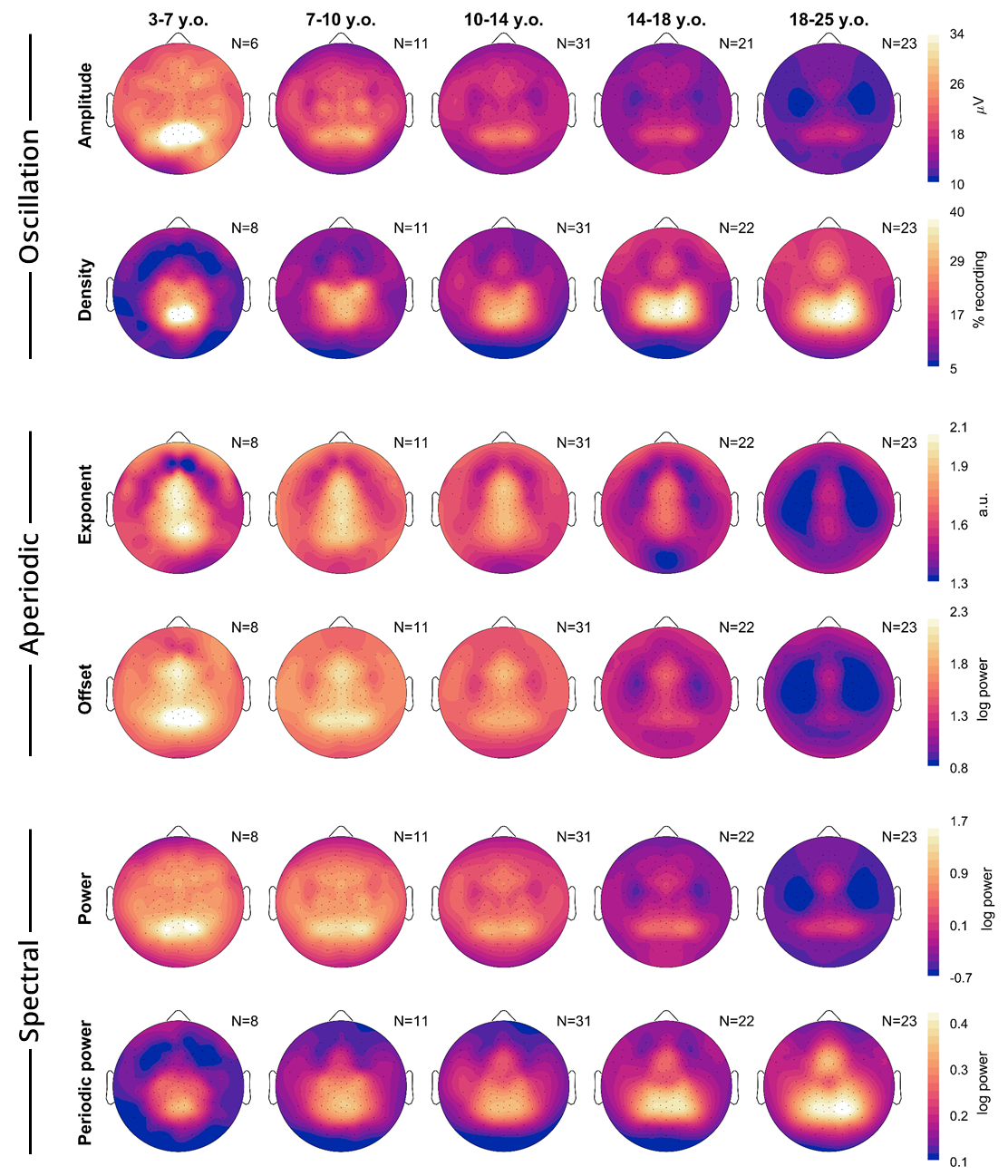


**Figure 4: Topography averages of wake EEG measures.** Each plot is a schematic of the EEG viewed from above, with the nose on top. Lighter colors indicate greater magnitude over a given location for that measure (rows). Only neurotypical participants and oddball recordings were included, and participants were grouped into age bins (columns). Multiple oddball recordings from different sessions and times of day were first averaged for each participant. The number of participants included is indicated in the top right corner of each plot. Acronyms: y.o., years-old; a.u., arbitrary units.

To determine the topography of overnight changes in EEG measures, we performed linear mixed effects models for each channel, with the model $Measure \sim Time + Task + (1|Participant) + (1|Participant:Session)$, dividing participants into 4 age bins (the youngest 3-7 were excluded as they were too few, with too few recordings). Figure 5 plots the β estimates for the effect of Time.

Amplitudes showed widespread overnight decreases across all age groups, however the decrease was largest in occipital channels for the youngest group, and slightly more fronto-temporal in young adults. The overnight density topographies resembled the average density topographies from Figure 4, in terms of location of the effects. The youngest group showed the largest overnight decrease in the same midline-occipital spot where there were the most oscillations (Figure 4), and adults showed the largest increase in the same bilateral occipital-parietal areas where they had the largest densities.

The overnight increase in the steepness of exponents was widespread, but peaked in an occipital spot in all age groups, with additional bilateral frontal spots in <18-year-olds. These topographies do not correspond to the average topography of exponents from Figure 4. Offsets revealed widespread decreases, with localized increases in the same occipital locations for which exponents increased the most. This suggests that aperiodic offsets generally decrease, although the increase in exponent steepness contrasts this effect.

Power and periodic power again showed similarities to amplitudes and densities respectively, however while densities increased in the 14–18-year-olds, periodic power decreased. Likewise, the overnight increase in periodic power for >18-year-olds was more occipital and lateral than the increase in densities, and the larger decrease in amplitudes in occipital regions of 7–10-year-olds was less evident in power than for amplitudes.


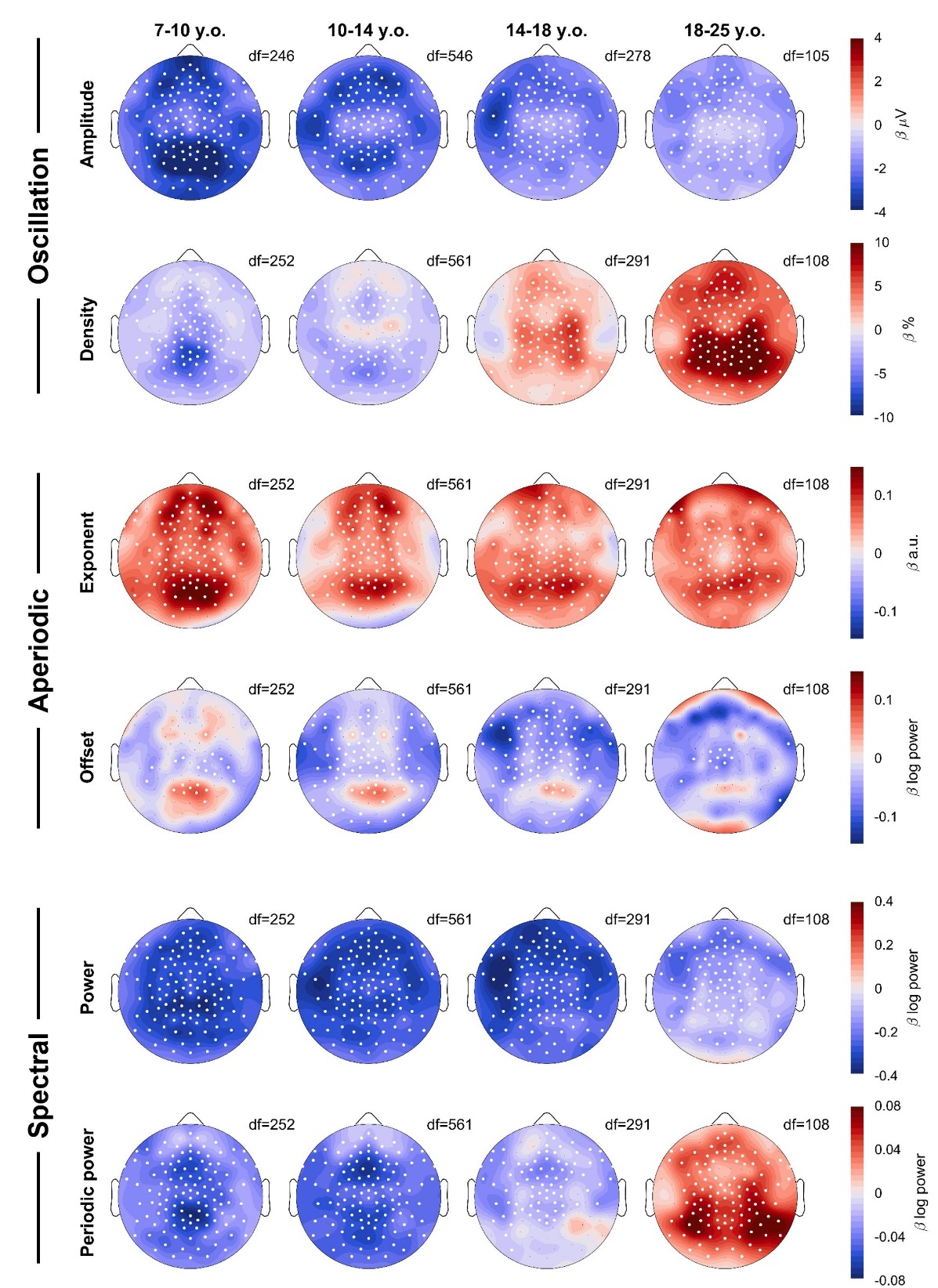


**Figure 5: Topographies of wake EEG overnight changes.** A linear mixed effects model was run for each measure, each age group, and each channel: Measure ~ Time + Task + (1|Participant) + (1|Participant:Session). Color reflects β estimates for the fixed effect *Time*, such that red indicates an overnight increase in that measure. The factor *Task* was not included for the 18-25 y.o. group, as these participants only performed oddballs. White dots indicate channels for which the β estimate was statistically significant, corrected for multiple comparisons with FDR (false discovery rate). Black dots indicate remaining channels. Data includes both patients and neurotypical controls. Degrees of freedom (df) are provided for each plot. For each column, the sample sizes were N = 29, 67, 36, & 23.

## Frequency-specific wake EEG changes with age and sleep pressure

In the previous analyses, we had pooled all frequencies between 4 and 16 Hz. Subsequently, we explored how oscillation and spectral EEG measures changed for each frequency, this time averaging channels (Suppl. Figure 6-1). We found that oscillations amplitudes and spectral power showed gradual changes across both age and frequency, whereas average density and periodic power had distinct peak values between 8 and 11 Hz, with the peak shifting upwards with age, a well-known property of alpha oscillations during development (Smith, 1938; Freschl et al., 2022; Tröndle et al., 2022). Overnight densities (and periodic power) showed decreases in higher frequencies (>11 Hz, i.e. low beta) and increases in alpha. These increases only began between 8-10 years of age, they were strongest in adults, and the range shifted to higher frequencies with age.

Given the dissociation between alpha and beta for density, we explored the topographic changes in density, split by both age and frequency band. In Figure 6, we plot average values. Theta oscillations were the overall rarest. They were most prevalent in the youngest children, and with age, the peak in theta density gradually shifted upward and decreased in magnitude. Alpha density instead started as a midline occipital cluster in the 3–7-year-olds. Alpha densities in the occipital spot decreased in the 7-10 cohort, with bilateral central spots instead becoming more pronounced. With age, these three peaks morphed into a continuous occipital-parietal cluster, while in the remaining channels overall density of alpha increased. Lastly, low beta oscillations showed yet another topography. They were almost completely absent in the youngest group, and started to appear in the 7–10-year-olds as lateral occipital peaks and a frontal midline spot. Gradually, the lateral peaks converge towards the midline, and in adults became partially overlapping with alpha, on average right-lateralized.


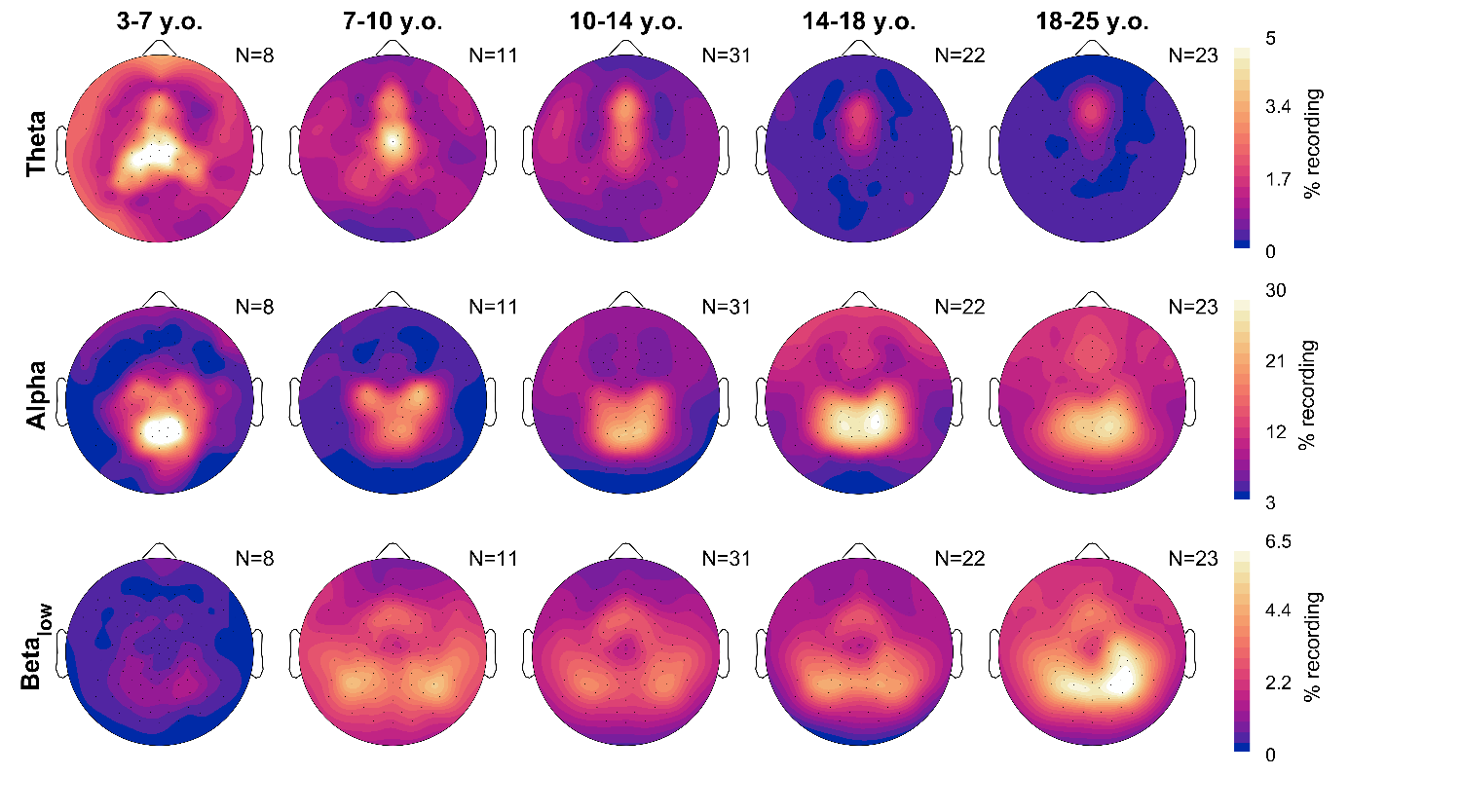


**Figure 6: Average topographies of oscillation densities, split by frequency band.** Recordings are the same as in Figure 4. Theta is 4-7 Hz, alpha is 8-11 Hz, and low beta is 12-16 Hz. Each band has a different color scale range.

The overnight changes in density split by frequency are in Figure 7, based on the model $Density \sim Time + Task + (1|Participant) + (1|Participant:Session)$. Like in Figure 5, these reflect the β estimates for the fixed effect of Time.

The overnight changes in theta density were small (around 1%), however quite variable by region and age. In the 7–10-year-olds, theta density generally increased overnight except in a central spot, exactly where the largest theta densities were seen in Figure 6, which instead decreased. This continued for the 10–14-year-olds. In the 14–18-year-olds, there were no significant effects, however the midline theta spot, now more frontal, showed a non-significant decrease. In adults, only some scattered theta increases were observed.

For alpha, the main occipital spot in the 7–10-year-olds decreased overnight. Already in the 10–14-year-olds a bilateral central alpha rhythm started to increase overnight, with still some slight decreases in the occipital spot. The overnight increases spread to the entire scalp in adolescents and adults, peaking in occipital parietal areas, especially right lateralized. For low beta, across all ages there were decreases, with the peak shifting across age bins.


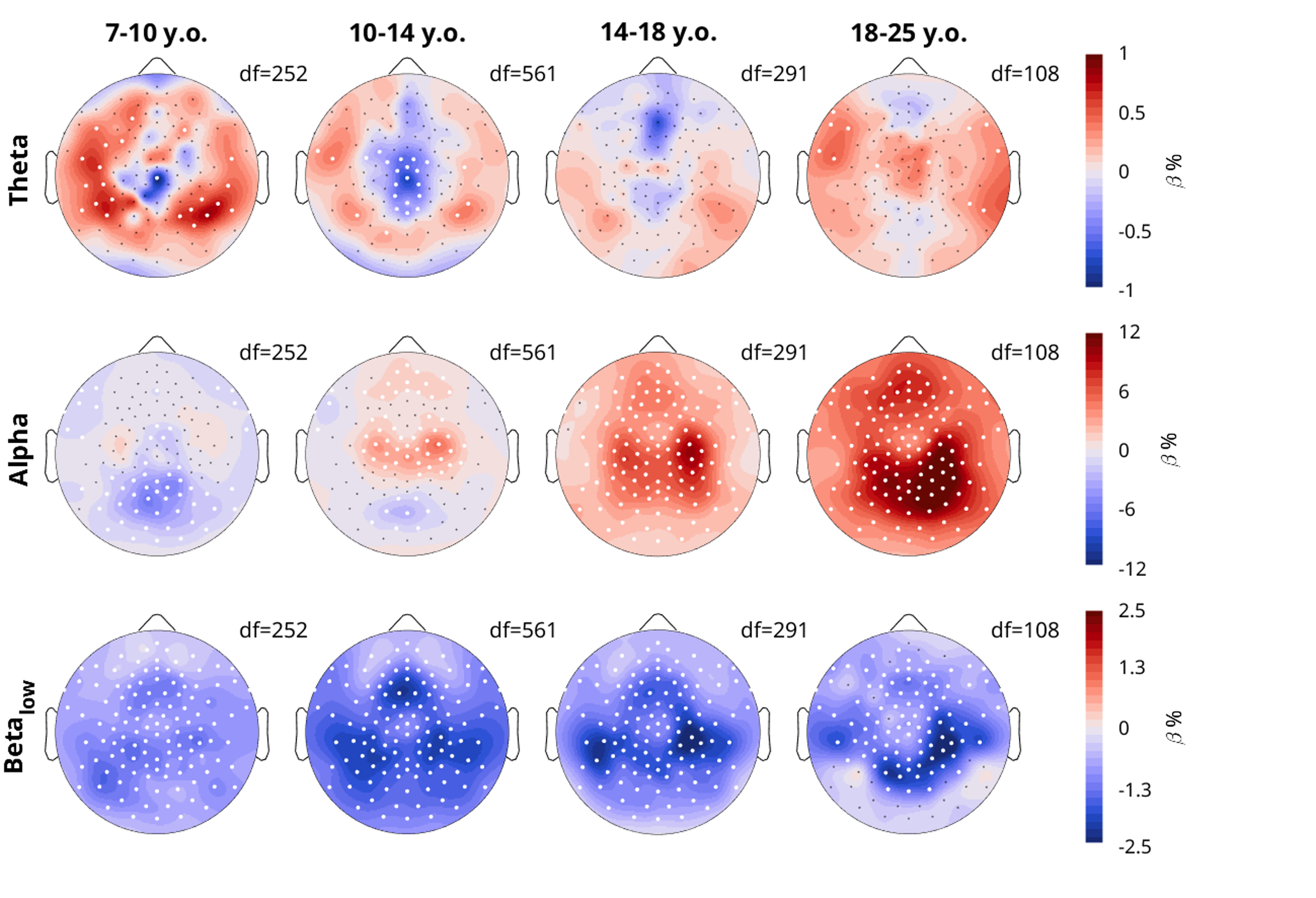


**Figure 7: Topographies of overnight density changes, split by frequency band**. Participants and plot information are the same as in Figure 5, with color indicating the β estimates for the linear mixed effects model, such that red indicates an overnight increase in density, white dots statistically significant channels, FDR corrected for multiple comparisons. The model was Density ~ Time + Task + (1|Participant) + (1|Participant:Session).

## Effect of ADHD on the wake EEG topography

In our initial mixed effects models pooling channels and frequencies, we found no significant effects for ADHD, which is why subsequent analyses and figures no longer included a Group effect. However, we nevertheless conducted mixed effects models to determine the effect of ADHD for each channel: $Measure \sim Task + Time*Age + Group + \left( 1 | \mathrm{Participant} \right)+ \left( 1 | Participant:Session \right)$. We found no significant effects when correcting for multiple comparisons (Figure 8). However, amplitudes were on average lower in participants with ADHD and frontal exponents were steeper compared to controls.


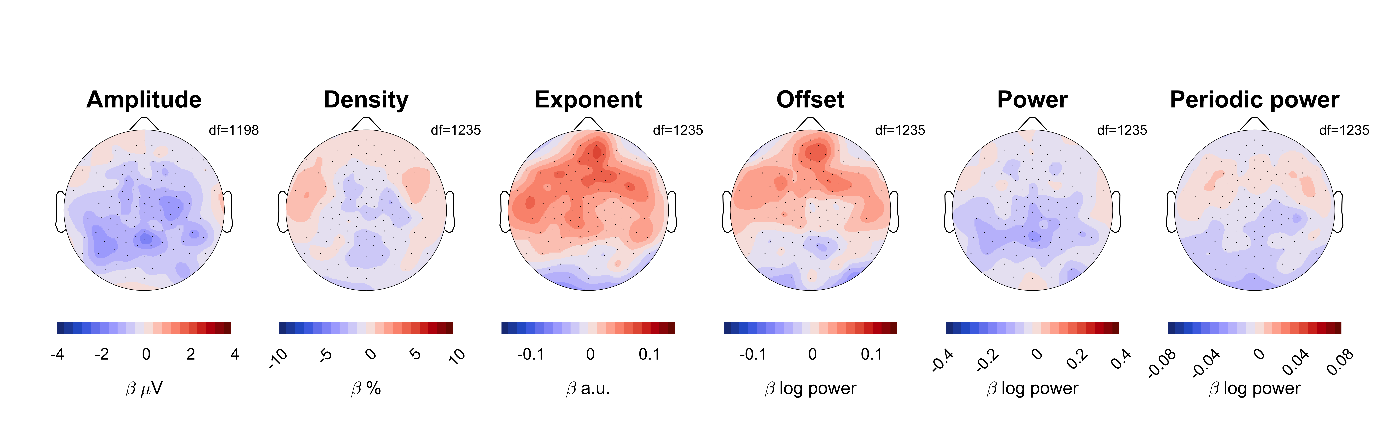


**Figure 8: Effects of ADHD on EEG measures.** Red indicates larger values in patients compared to controls. The scale for each topography is the same as for Figure 5. The model was Measure ~ Time*Age + Task + Group + (1|Participant) + (1|Participant:Session). White dots would have indicated statistically significant channels, following FDR correction for multiple comparisons.

# Discussion

In this study, we compared four distinct wake EEG measures and their relationship to development, recording time, and ADHD. Specifically, we hypothesized that oscillation amplitudes would behave like sleep slow waves, because both measures likely reflect neuronal synchronization due to synaptic density and plasticity. Our predictions were met on all accounts except for the sensitivity of wake amplitudes to ADHD. Of the four measures, only amplitudes decreased overnight in all ages *and* the overnight decrease was largest in younger children (Figure 3). Aperiodic offsets showed similar development and overnight changes, but they did not display larger overnight decreases in younger children, a key indicator of higher neural plasticity. Although overnight changes could reflect circadian influences, our previous wake EEG findings during sleep deprivation (Snipes et al., 2023) provide converging evidence that wake oscillation amplitudes are modulated by sleep pressure.

While oscillation amplitudes were the only wake measure that followed all the patterns expected for sleep pressure, every EEG measure reflected development. Average amplitudes, exponents, and offsets were all strongly anticorrelated with age, with offsets showing the strongest relationship (Figure 3). The correlation between average densities and age was weak, but we found distinctive regional patterns across age groups (Figure 6). Overnight changes in both oscillation amplitudes and density were robustly correlated with age (Figure 3, Figure 7), whereas overnight changes in exponents and offsets were not as affected by age, as evidenced by the low correlations in Figure 3. Finally, no measure showed significant effects of ADHD (Figure 8).

## Oscillation amplitudes

Like for slow waves during sleep, the decrease in wake oscillation amplitudes with age could be explained by decreasing synaptic density in the cortex across adolescence (Huttenlocher, 1979). The overnight decrease in wake amplitudes could be explained by sleep’s role in reducing net synaptic strength (Tononi and Cirelli, 2014; Cirelli and Tononi, 2022), and such plastic changes are more pronounced in children than adults (Jaramillo et al., 2020). The larger decrease in occipital regions in children compared to adults may be because primary sensory and motor areas obtain peak cortical thickness earlier in children, followed by adjacent secondary areas and finally frontal association areas (Shaw et al., 2008), resulting in an overall posterior-anterior maturation trajectory also for slow wave activity (Kurth et al., 2010). Therefore, younger children show larger overnight decreases in amplitudes in occipital areas because these areas undergo higher plastic changes at that maturational stage. Finally, even the significant sex difference was comparable to sleep slow wave activity (Mourtazaev et al., 1995), with wake oscillation amplitudes higher in females than males (although the effect was relatively small). This effect could be due to females’ smaller heads and thinner skulls (Dijk et al., 1989). However, Schaworonkow and Voytek (2021) did not find any changes in wake oscillation amplitudes from 1-7 month-old infants, despite this period corresponding to the fastest growth in head circumference (Roche et al., 1986). Therefore, it is unlikely that the more modest increases in head size from childhood to adolescence can entirely explain the large decrease in amplitudes we observed here.

While wake oscillation amplitudes behave largely like sleep slow waves, the effects are reduced. In adults the overnight decrease in wake amplitudes was near 0 μV (Figure 3), which is not the case for either slow wave slopes or amplitudes during sleep (Riedner et al., 2007; Jaramillo et al., 2020). One possible explanation for this is that the evening wake recordings fell within the wake maintenance zone. This is a circadian time window just before bedtime characterized by increased alertness (Strogatz et al., 1987; Shekleton et al., 2013). Oscillation amplitudes are significantly reduced in this window, counteracting the otherwise monotonic buildup in amplitudes that occurred throughout the day (Snipes et al., 2023). In adults the contrasting effect of the wake maintenance zone may be sufficient to equalize evening and morning amplitudes. Overall, wake oscillation amplitudes may reflect the same information as sleep slow waves but may be more affected by circadian or other factors. Therefore, wake amplitudes may be affected by the same underlying neuronal changes as sleep slow wave activity, but they are not sufficiently specific nor sensitive to replace this gold standard measure of sleep pressure.

## Oscillation densities

As could be expected, the densities of oscillations were the most complex EEG measure, with effects differing depending on age, time, topography, and oscillation frequency.

Theta oscillations were most prevalent in early childhood, and decreased progressively with age, supporting previous results measuring relative theta power (Somsen et al., 1997). We further found that the peak in theta densities steadily drifted more frontally across childhood and adolescence. This frontal theta in adults is known to originate from the midline prefrontal cortex, and to be anti-correlated to the default mode network (Scheeringa et al., 2008; Michels et al., 2010; Ishii et al., 2014). Therefore, this drift in theta may reflect the steady maturation of frontal cortices and the default mode network in particular (Fan et al., 2021).

### Theta

There is still unresolved contradictory evidence on the role of theta in adults (Snipes et al., 2022), without including the question of theta during development. On the one hand, theta is often associated with cognitive effort (Buzsáki, 2005; Mitchell et al., 2008; Cavanagh and Frank, 2014; Meyer et al., 2019). On the other, it is also associated with sleepiness (Smith, 1938; Finelli et al., 2000; Snipes et al., 2022) and fatigue (Wascher et al., 2014; Tran et al., 2020; Arnau et al., 2021). A possible resolution to this paradox is that there are distinct oscillations that originate from different circuits with different functions and just happen to occur at the same frequency. Our results in Figure 7 would support this, as the peak source of theta shows overnight *decreases*, whereas theta from the rest of the cortex instead shows overnight increases, even as the theta peak drifts more frontally. Alternatively, theta could reflect a general form of “idling rhythm” (Scheeringa et al., 2008; Snipes et al., 2022, 2024), originating from disengaged cortical areas, and what changes from evening to morning is which circuits tend to idle. This would make theta functionally comparable to alpha (Laufs et al., 2003), differing only by source and frequency.

### Alpha

Alpha begins in young childhood as a midline spot (Figure 6). Two lateral central peaks become more defined at 7-10 years of age. These likely reflect sensorimotor mu rhythms which appear when motor activity is absent or even suppressed (Pineda, 2005; Pfurtscheller et al., 2006), and is already present in infants (Berchicci et al., 2011). We find that with age, they become topographically indistinguishable from occipital alpha, at least when recorded during an oddball task. These lateral central peaks are the first to show overnight increases in 10-14 year-olds, while the overnight decrease in the occipital midline spot becomes less prominent. The overnight increase in alpha densities then spreads over bilateral parietal and occipital areas across adolescence and adulthood. This dissociation between overnight decreases in childhood and increases in adulthood, as well as the slight differences in topography, could suggest that occipital alpha is in fact qualitatively distinct in children and adults. However, these rhythms were previously considered functionally equivalent because also in infants alpha power increases with eyes closed compared to eyes open (Stroganova et al., 1999). More research is needed on the sources of these oscillations.

It is also possible that this dissociation in overnight density changes is driven by some other difference with age, such as a longer window of sleep inertia in young children, longer sleep duration, or a shifted circadian rhythm compared to adults. Melatonin in the morning is elevated in children under 10, whereas older children and adolescents have morning melatonin levels comparable with the rest of the day (Attanasio et al., 1985). In adults, alpha power fluctuates with circadian rhythm and is therefore synchronized to melatonin levels (Cajochen et al., 2002). Therefore, it is possible that the dissociation of decreasing/increasing alpha densities originates from children and adults being at different phases of their alpha circadian rhythm in the morning. More research is needed into the circadian effects on the EEG across development.

### Beta

Finally, we found overnight decreases in low beta densities across ages. There is ample literature on “beta bursts” from sensorimotor areas (Jones et al., 2009; Feingold et al., 2015; Shin et al., 2017; Little et al., 2019; Wessel, 2020; Lundqvist et al., 2024), however these are likely qualitatively distinct from the beta oscillations we observed. First, the topography of sensorimotor beta rhythms was found to be either bilateral-central in infants or frontal in adults (Rayson et al., 2023), unlike the bilateral occipital topographies in Figure 6. Second, sensorimotor beta was found to occur in “bursts” of power less than 150 ms long (Sherman et al., 2016) and could thus correspond to only 1-3 cycles of beta (Jones, 2016; van Ede et al., 2018; Rayson et al., 2023). We detected bursts that were *at least* 3 cycles long, only up to 16 Hz, and therefore always longer than 187 ms. Therefore, the low-beta bursts we captured with cycle-by-cycle analysis are likely distinct from the previously investigated sensorimotor beta activity. It remains an open question what is the functional role of these low-beta oscillations, and why they may be less common the morning after sleep.

## Aperiodic offsets and exponents

Our results on offsets and exponents replicate previous findings: they decrease linearly with age (Cellier et al., 2021; Hill et al., 2022; Tröndle et al., 2022) and originate from broad, primarily midline sources (Favaro et al., 2023). As in Lendner et al. (2023), we found exponents becoming increasingly steeper after sleep, extending this finding across ages. We further found, unusually, that exponents and offsets change in opposite directions, with offsets decreasing after a night of sleep. For aperiodic activity measured *during* sleep, both exponents and offsets decrease across the night, and the decrease decreases with age (Horváth et al., 2022). This indicates that changes in aperiodic activity related to sleep-wake history do not reflect the same information when measured during wake or during sleep. It is possible that during sleep the decrease in exponents reflects the decrease in sleep pressure, whereas during wake the increase in exponents could reflect something like lingering sleep inertia in the morning. Notably, the change in aperiodic exponents with age and sleep depth affect primarily lower frequencies (Favaro et al., 2023), whereas the overnight wake EEG changes affect primarily higher frequencies (Figure 2). This means that the pivot point (the frequency at which the aperiodic signal rotates) of the exponent differs, and this likely has biological significance. Future studies should, as in Bódizs et al. (2021), specifically identify such pivot points, also to better dissociate changes in offset and exponent.

Second to oscillation amplitudes, aperiodic offsets showed the closest resemblance to sleep slow wave activity, decreasing with age and decreasing overnight, In line with increases in offsets following sleep deprivation (Bai et al., 2024). However, offsets did not show the characteristic decrease in overnight changes with age that was observed for sleep slow waves and wake amplitudes (Figure 3). This could suggest that offsets also reflect changes in synaptic strength and plasticity, but may be less sensitive or specific than wake amplitudes, especially to overnight changes.

## Spectral power vs oscillation burst detection

As explained by previous papers, changes in spectral power do not differentiate between changes in oscillation amplitudes or densities (Quinn et al., 2019; Tal et al., 2020; Zich et al., 2020; Donoghue et al., 2022; Snipes et al., 2023). With this study, we again demonstrate that amplitudes and densities meaningfully change independently, both across sleep and development. However, we also found that the changes in amplitude were largely captured by changes in average power, whereas changes in density were largely captured by changes in periodic power. This suggests that power and periodic power can be used as proxies for amplitudes and densities. Previous studies of development have likewise found differences between power and *relative power* (similar to periodic power) across development (Somsen et al., 1997). These results may be re-interpreted as the dissociation between changes in amplitude and density.

Arguing against this approach, however, is the topography of overnight changes in 14–18-year-olds: oscillation densities increased overnight, whereas periodic power decreased, likely reflecting the greater influence of the overnight decrease in amplitudes. Similarly, the overnight increase in densities is more central than the overnight increase in periodic power. Therefore, differences between power and periodic power may suggest different influences of oscillation amplitudes and densities, but to know for certain, oscillations should be measured directly.

## ADHD

Despite a relatively large sample size (N = 58), we did not observe any significant effects of ADHD on our EEG measures. We did find exponents to be steeper on average in patients, supporting the results of Robertson et al. (2019), and contrasting those of Ostlund et al. (2021). One explanation could be that our participants were performing tasks for most recordings, and the differences between patients and controls may mostly emerge in resting EEG. It’s also possible our analysis did not reach significance because our participants were a combination of both medicated and unmedicated patients, and it is known that medication will reduce the effects of ADHD on the EEG (Furrer et al., 2019; Karalunas et al., 2022).

Another important difference with previous studies is that our participants were screened for habitual good sleep quality. This was originally intended to ensure both groups had comparable sleep in the laboratory. However, generally around 40-55% of children with ADHD report sleep deficits (Corkum et al., 1998; Holmberg and Hjern, 2006; Konofal et al., 2010; Becker et al., 2019; Xian et al., 2025), so it is possible that poor sleep quality in patients contributed to differences in the wake EEG observed in other studies (Clarke et al., 2020; Pinggal et al., 2026). In other words, because of habitual lower sleep quality and increased daytime sleepiness, patients with ADHD could have presented with higher theta or steeper exponents compared to controls, but this effect is not specific to the pathology of ADHD.

Regardless of the reason, given that we do not see any systematic differences between patients and controls, none of the wake EEG measures we tested make for a reliable intrinsic marker of ADHD which could potentially be used to aid diagnosis. Instead, research investigating such markers in these and other patient populations should take special care to control for sleep/wake history and sleep quality, as these may have a greater impact on the EEG. Instead, within the same participants we observed significant differences in sleep slow wave activity from controls (Furrer et al., 2019), suggesting sleep is more sensitive to developmental deficits.

## Limitations

The primary limitation of this study is its inability to disentangle the effects of sleep pressure from those of circadian rhythms. To do so would require substantially more intensive protocols, such as sleep deprivation, sleep restriction, or shifting sleep windows over several days. However, future studies could collect more wake recordings throughout the day to provide an indication as to whether the changes in oscillations amplitude are circadian or homeostatic (or both).

Another important limitation of this study is the scarcity of datasets under 8. It is known that in young children there is a switch from primarily synaptic growth to primarily synaptic pruning, peaking in different cell populations and regions at different ages (Shaw et al., 2008; Petanjek et al., 2011; Cao et al., 2020), which is also reflected in sleep slow wave activity peaking around this age (Feinberg and Campbell, 2013). Wilkinson et al. (2024) found an increase with age in both offsets and exponents across infants 0-3 years old, and McSweeney et al. (2023) found a quadratic relationship between age and offsets/exponents, such that they peaked at 5-7 years old (see also (Antúnez et al., 2025)). This would suggest more complex relationships between age and EEG measures than the linear trends observed here.

A further limitation of the data is its lack of uniformity, pooling multiple experimental paradigms and tasks, and medicated and unmedicated patients. Our primary findings remain significant also for more uniform subsets of the data (e.g. Figure 3 is only oddball), but it’s possible that different tasks would generate different topographies, and significant effects of ADHD would have emerged from entirely unmedicated patients. Future studies may find significant effects by using strictly unmedicated patients, or carefully controlling treatment.

Additionally, if one assumes that aperiodic and oscillatory signals are a linear sum of each other in the time-domain of the EEG signal, it is possible that the aperiodic changes explain part of the changes in oscillation amplitudes. A better understanding of the relationship between oscillations and the aperiodic signal are needed to validate such an assumption, and then precise simulations would be needed to determine the magnitude of this effect. However, in practice we have demonstrated that oscillation amplitudes can behave differently from aperiodic measures, and therefore it is at least useful to measure them independently.

Finally, our data is limited to EEG. Future studies and analyses would greatly benefit by comparing these measures to structural and functional brain changes observable with MRI, as well as cognitive and behavioral outcome measures related to development. This would bridge the gap between a purely basic research finding to practical applications.

## Conclusions

We have found that overnight changes in wake oscillations provide distinct markers of brain maturation. Both absolute amplitudes and overnight changes in amplitudes decrease linearly with age, the effect more occipital in younger children. Overnight changes in oscillation density, especially of alpha oscillations, dissociate children from adolescents and adults by switching from an overnight decrease to an increase in density. Understanding the reason behind this effect would likely provide important information on brain development around puberty and adolescence. At the same time, these strong interactions can become confounds in studies where sleep/wake history and circadian rhythms differ. Both sleep and wake research should take into consideration the dynamics of EEG across time, especially in young populations.

# References

Antúnez M, McSweeney M, Zeytinoglu S, Tan E, Zeanah CH, Nelson CA, Fox NA (2025) Exploring background aperiodic electroencephalography (EEG) activity in the Bucharest Early Intervention Project. Dev Psychol 61:1371–1383.

Arnau S, Brümmer T, Liegel N, Wascher E (2021) Inverse effects of time-on-task in task-related and task-unrelated theta activity. Psychophysiology 58:e13805.

Attanasio A, Borrelli P, Gupta D (1985) Circadian rhythms in serum melatonin from infancy to adolescence. J Clin Endocrinol Metab 61:388–390.

Bai D, Hu J, Jülich S, Lei X (2024) Impact of sleep deprivation on aperiodic activity: a resting-state EEG study. J Neurophysiol 132:1577–1588.

Bazanova OM, Vernon D (2014) Interpreting EEG alpha activity. Neurosci Biobehav Rev, Applied Neuroscience: Models, methods, theories, reviews. A Society of Applied Neuroscience (SAN) special issue. 44:94–110.

Becker SP, Langberg JM, Eadeh H-M, Isaacson PA, Bourchtein E (2019) Sleep and daytime sleepiness in adolescents with and without ADHD: differences across ratings, daily diary, and actigraphy. J Child Psychol Psychiatry 60:1021–1031.

Benjamini Y, Yekutieli D (2001) The Control of the False Discovery Rate in Multiple Testing under Dependency. Ann Stat 29:1165–1188.

Berchicci M, Zhang T, Romero L, Peters A, Annett R, Teuscher U, Bertollo M, Okada Y, Stephen J, Comani S (2011) Development of Mu Rhythm in Infants and Preschool Children. Dev Neurosci 33:130–143.

Bódizs R, Schneider B, Ujma PP, Horváth CG, Dresler M, Rosenblum Y (2024) Fundamentals of sleep regulation: Model and benchmark values for fractal and oscillatory neurodynamics. Prog Neurobiol 234:102589.

Bódizs R, Szalárdy O, Horváth C, Ujma PP, Gombos F, Simor P, Pótári A, Zeising M, Steiger A, Dresler M (2021) A set of composite, non-redundant EEG measures of NREM sleep based on the power law scaling of the Fourier spectrum. Sci Rep 11:2041.

Borbély AA (1982) A two process model of sleep regulation. Hum Neurobiol 1:195–204.

Buchmann A, Ringli M, Kurth S, Schaerer M, Geiger A, Jenni OG, Huber R (2011) EEG Sleep Slow-Wave Activity as a Mirror of Cortical Maturation. Cereb Cortex 21:607–615.

Buzsáki G (2005) Theta rhythm of navigation: Link between path integration and landmark navigation, episodic and semantic memory. Hippocampus 15:827–840.

Cajochen C, Wyatt JK, Czeisler CA, Dijk DJ (2002) Separation of circadian and wake duration-dependent modulation of EEG activation during wakefulness. Neuroscience 114:1047–1060.

Campbell IG, Feinberg I (2009) Longitudinal trajectories of non-rapid eye movement delta and theta EEG as indicators of adolescent brain maturation. Proc Natl Acad Sci U S A 106.

Cao J, Herman AB, West GB, Poe G, Savage VM (2020) Unraveling why we sleep: Quantitative analysis reveals abrupt transition from neural reorganization to repair in early development. Sci Adv 6:eaba0398.

Cavanagh JF, Frank MJ (2014) Frontal theta as a mechanism for cognitive control. Trends Cogn Sci 18:414–421.

Cellier D, Riddle J, Petersen I, Hwang K (2021) The development of theta and alpha neural oscillations from ages 3 to 24 years. Dev Cogn Neurosci 50:100969.

Cirelli C, Tononi G (2022) The why and how of sleep-dependent synaptic down-selection. Semin Cell Dev Biol, Special Issue: Interplay between non-canonical and canonical Wnt signalling by Terry Van Raay/ Special Issue: The making of memories by Patricio Opazo and Victor Anggono 125:91–100.

Clarke AR, Barry RJ, Johnstone S (2020) Resting state EEG power research in Attention-Deficit/Hyperactivity Disorder: A review update. Clin Neurophysiol 131:1463–1479.

Cole S, Voytek B (2019) Cycle-by-cycle analysis of neural oscillations. J Neurophysiol 122.

Corkum P, Tannock R, Moldofsky H (1998) Sleep Disturbances in Children With Attention‐Deficit/Hyperactivity Disorder. J Am Acad Child Adolesc Psychiatry 37:637–646.

Delorme A, Makeig S (2004) EEGLAB: An open source toolbox for analysis of single-trial EEG dynamics including independent component analysis. J Neurosci Methods 134:9–21.

Dijk DJ, Beersma DGM, Bloem GM (1989) Sex Differences in the Sleep EEG of Young Adults: Visual Scoring and Spectral Analysis. Sleep 12:500–507.

Dijk DJ, Beersma DGM, Daan S (1987) EEG Power Density during Nap Sleep: Reflection of an Hourglass Measuring the Duration of Prior Wakefulness. J Biol Rhythms 2:207–219.

Donoghue T, Haller M, Peterson EJ, Varma P, Sebastian P, Gao R, Noto T, Lara AH, Wallis JD, Knight RT, Shestyuk A, Voytek B (2020) Parameterizing neural power spectra into periodic and aperiodic components. Nat Neurosci 23:1655–1665.

Donoghue T, Schaworonkow N, Voytek B (2022) Methodological considerations for studying neural oscillations. Eur J Neurosci 55:3502–3527.

Fan F, Liao X, Lei T, Zhao T, Xia M, Men W, Wang Y, Hu M, Liu J, Qin S, Tan S, Gao J-H, Dong Q, Tao S, He Y (2021) Development of the default-mode network during childhood and adolescence: A longitudinal resting-state fMRI study. NeuroImage 226:117581.

Fattinger S, Kurth S, Ringli M, Jenni OG, Huber R (2017) Theta waves in children’s waking electroencephalogram resemble local aspects of sleep during wakefulness. Sci Rep 7:11187.

Favaro J, Colombo MA, Mikulan E, Sartori S, Nosadini M, Pelizza MF, Rosanova M, Sarasso S, Massimini M, Toldo I (2023) The maturation of aperiodic EEG activity across development reveals a progressive differentiation of wakefulness from sleep. NeuroImage 277:120264.

Feinberg I, Campbell IG (2013) Longitudinal sleep EEG trajectories indicate complex patterns of adolescent brain maturation. Am J Physiol Regul Integr Comp Physiol 304:R296-303.

Feingold J, Gibson DJ, DePasquale B, Graybiel AM (2015) Bursts of beta oscillation differentiate postperformance activity in the striatum and motor cortex of monkeys performing movement tasks. Proc Natl Acad Sci 112:13687–13692.

Finelli LA, Baumann H, Borbély AA, Achermann P (2000) Dual electroencephalogram markers of human sleep homeostasis: Correlation between theta activity in waking and slow-wave activity in sleep. Neuroscience 101:523–529.

Freschl J, Azizi LA, Balboa L, Kaldy Z, Blaser E (2022) The development of peak alpha frequency from infancy to adolescence and its role in visual temporal processing: A meta-analysis. Dev Cogn Neurosci 57:101146.

Furrer M, Jaramillo V, Volk C, Ringli M, Aellen R, Wehrle FM, Pugin F, Kurth S, Brandeis D, Schmid M, Jenni OG, Huber R (2019) Sleep EEG slow-wave activity in medicated and unmedicated children and adolescents with attention-deficit/hyperactivity disorder. Transl Psychiatry 9.

Furrer M, Ringli M, Kurth S, Brandeis D, Jenni OG, Huber R (2020) The experience-dependent increase in deep sleep activity is reduced in children with attention-deficit/hyperactivity disorder. Sleep Med 75.

Ghilardi M-F, Ghez C, Dhawan V, Moeller J, Mentis M, Nakamura T, Antonini A, Eidelberg D (2000) Patterns of regional brain activation associated with different forms of motor learning. Brain Res 871:127–145.

Hill AT, Clark GM, Bigelow FJ, Lum JAG, Enticott PG (2022) Periodic and aperiodic neural activity displays age-dependent changes across early-to-middle childhood. Dev Cogn Neurosci 54:101076.

Holmberg K, Hjern A (2006) Health complaints in children with attention-deficit/hyperactivity disorder. Acta Paediatr Int J Paediatr 95.

Horváth CG, Szalárdy O, Ujma PP, Simor P, Gombos F, Kovács I, Dresler M, Bódizs R (2022) Overnight dynamics in scale-free and oscillatory spectral parameters of NREM sleep EEG. Sci Rep 12:18409.

Huttenlocher PR (1979) Synaptic density in human frontal cortex - developmental changes and effects of aging. Brain Res 163:195–205.

Ishii R, Canuet L, Ishihara T, Aoki Y, Ikeda S, Hata M, Katsimichas T, Gunji A, Takahashi H, Nakahachi T, Iwase M, Takeda M (2014) Frontal midline theta rhythm and gamma power changes during focused attention on mental calculation: An MEG beamformer analysis. Front Hum Neurosci 8.

Jaramillo V, Volk C, Maric A, Furrer M, Fattinger S, Kurth S, Lustenberger C, Huber R (2020) Characterization of overnight slow-wave slope changes across development in an age-, amplitude-, and region-dependent manner. Sleep 43.

Jenni OG, Carskadon MA (2004) Spectral Analysis of the Sleep Electroencephalogram During Adolescence. Sleep 27:774–783.

Jones SR (2016) When brain rhythms aren’t “rhythmic”: implication for their mechanisms and meaning. Curr Opin Neurobiol 40:72–80.

Jones SR, Pritchett DL, Sikora MA, Stufflebeam SM, Hämäläinen M, Moore CI (2009) Quantitative Analysis and Biophysically Realistic Neural Modeling of the MEG Mu Rhythm: Rhythmogenesis and Modulation of Sensory-Evoked Responses. J Neurophysiol 102:3554–3572.

Karalunas SL, Ostlund BD, Alperin BR, Figuracion M, Gustafsson HC, Deming EM, Foti D, Antovich D, Dude J, Nigg J, Sullivan E (2022) Electroencephalogram aperiodic power spectral slope can be reliably measured and predicts ADHD risk in early development. Dev Psychobiol 64:e22228.

Klimesch W (1999) EEG alpha and theta oscillations reflect cognitive and memory performance: a review and analysis. Brain Res Brain Res Rev 29:169–195.

Konofal E, Lecendreux M, Cortese S (2010) Sleep and ADHD. Sleep Med 11.

Korotchikova I, Connolly S, Ryan CA, Murray DM, Temko A, Greene BR, Boylan GB (2009) EEG in the healthy term newborn within 12hours of birth. Clin Neurophysiol 120:1046–1053.

Kurth S, Ringli M, Geiger A, LeBourgeois M, Jenni OG, Huber R (2010) Mapping of cortical activity in the first two decades of life: A high-density sleep electroencephalogram study. J Neurosci 30.

Laufs H, Kleinschmidt A, Beyerle A, Eger E, Salek-Haddadi A, Preibisch C, Krakow K (2003) EEG-correlated fMRI of human alpha activity. NeuroImage 19:1463–1476.

Le Bon-Jego M, Yuste R (2007) Persistently active, pacemaker-like neurons in neocortex. Front Neurosci 1.

Lee IA, Preacher KJ (2013) Calculation for the test of the difference between two dependent correlations with one variable in common [Computer software]. Available from http://quantpsy.org.

Lendner JD, Helfrich RF, Mander BA, Romundstad L, Lin JJ, Walker MP, Larsson PG, Knight RT (2020) An electrophysiological marker of arousal level in humans. eLife 9:e55092.

Lendner JD, Niethard N, Mander BA, van Schalkwijk FJ, Schuh-Hofer S, Schmidt H, Knight RT, Born J, Walker MP, Lin JJ, Helfrich RF (2023) Human REM sleep recalibrates neural activity in support of memory formation. Sci Adv 9:eadj1895.

Little S, Bonaiuto J, Barnes G, Bestmann S (2019) Human motor cortical beta bursts relate to movement planning and response errors. PLOS Biol 17:e3000479.

Lundqvist M, Miller EK, Nordmark J, Liljefors J, Herman P (2024) Beta: bursts of cognition. Trends Cogn Sci 0.

McSweeney M, Morales S, Valadez EA, Buzzell GA, Yoder L, Fifer WP, Pini N, Shuffrey LC, Elliott AJ, Isler JR, Fox NA (2023) Age-related trends in aperiodic EEG activity and alpha oscillations during early- to middle-childhood. NeuroImage 269:119925.

Meyer M, Endedijk HM, van Ede F, Hunnius S (2019) Theta oscillations in 4-year-olds are sensitive to task engagement and task demands. Sci Rep 9:6049.

Michels L, Bucher K, Lüchinger R, Klaver P, Martin E, Jeanmonod D, Brandeis D (2010) Simultaneous EEG-fMRI during a working memory task: Modulations in low and high frequency bands. PLoS ONE 5.

Mitchell DJ, McNaughton N, Flanagan D, Kirk IJ (2008) Frontal-midline theta from the perspective of hippocampal “theta.” Prog Neurobiol 86:156–185.

Mourtazaev MS, Kemp B, Zwinderman AH, Kamphuisen HAC (1995) Age and Gender Affect Different Characteristics of Slow Waves in the Sleep EEG. Sleep 18:557–564.

Ostlund BD, Alperin BR, Drew T, Karalunas SL (2021) Behavioral and cognitive correlates of the aperiodic (1/f-like) exponent of the EEG power spectrum in adolescents with and without ADHD. Dev Cogn Neurosci 48:100931.

Ostlund BD, Donoghue T, Anaya B, Gunther KE, Karalunas SL, Voytek B, Pérez-Edgar KE (2022) Spectral parameterization for studying neurodevelopment: How and why. Dev Cogn Neurosci 54:101073.

Perkel DH, Schulman JH, Bullock TH, Moore GP, Segundo JP (1964) Pacemaker Neurons: Effects of Regularly Spaced Synaptic Input. Science 145:61–63.

Pernet C, Garrido MI, Gramfort A, Maurits N, Michel CM, Pang E, Salmelin R, Schoffelen JM, Valdes-Sosa PA, Puce A (2020) Issues and recommendations from the OHBM COBIDAS MEEG committee for reproducible EEG and MEG research. Nat Neurosci 23:1473–1483.

Petanjek Z, Judaš M, Šimić G, Rašin MR, Uylings HBM, Rakic P, Kostović I (2011) Extraordinary neoteny of synaptic spines in the human prefrontal cortex. Proc Natl Acad Sci 108:13281–13286.

Pfurtscheller G, Brunner C, Schlögl A, Lopes da Silva FH (2006) Mu rhythm (de)synchronization and EEG single-trial classification of different motor imagery tasks. NeuroImage 31:153–159.

Pineda JA (2005) The functional significance of mu rhythms: Translating “seeing” and “hearing” into “doing.” Brain Res Rev 50:57–68.

Pinggal E, Jackson J, Kusztor A, Chapman D, Windt J, Drummond SPA, Silk TJ, Bellgrove MA, Andrillon T (2026) Sleep-like Slow Waves During Wakefulness Mediate Attention and Vigilance Difficulties in Adult Attention-Deficit/Hyperactivity Disorder. J Neurosci.

Quinn AJ, van Ede F, Brookes MJ, Heideman SG, Nowak M, Seedat ZA, Vidaurre D, Zich C, Nobre AC, Woolrich MW (2019) Unpacking Transient Event Dynamics in Electrophysiological Power Spectra. Brain Topogr 32:1020–1034.

Rayson H, Szul MJ, El-Khoueiry P, Debnath R, Gautier-Martins M, Ferrari PF, Fox N, Bonaiuto JJ (2023) Bursting with Potential: How Sensorimotor Beta Bursts Develop from Infancy to Adulthood. J Neurosci 43:8487–8503.

Riedner BA, Vyazovskiy VV, Huber R, Massimini M, Esser S, Murphy M, Tononi G (2007) Sleep homeostasis and cortical synchronization: III. A high-density EEG study of sleep slow waves in humans. Sleep 30:1643–1657.

Ringli M, Souissi S, Kurth S, Brandeis D, Jenni OG, Huber R (2013) Topography of sleep slow wave activity in children with attention-deficit/hyperactivity disorder. Cortex 49:340–347.

Robertson MM, Furlong S, Voytek B, Donoghue T, Boettiger CA, Sheridan MA (2019) EEG power spectral slope differs by ADHD status and stimulant medication exposure in early childhood. J Neurophysiol 122:2427–2437.

Roche AF, Mukherjee D, Guo S (1986) Head Circumference Growth Patterns: Birth to 18 Years. Hum Biol 58:893–906.

Schaworonkow N, Voytek B (2021) Longitudinal changes in aperiodic and periodic activity in electrophysiological recordings in the first seven months of life. Dev Cogn Neurosci 47:100895.

Scheeringa R, Bastiaansen MCM, Petersson KM, Oostenveld R, Norris DG, Hagoort P (2008) Frontal theta EEG activity correlates negatively with the default mode network in resting state. Int J Psychophysiol 67:242–251.

Schneider B, Szalárdy O, Ujma PP, Simor P, Gombos F, Kovács I, Dresler M, Bódizs R (2022) Scale-free and oscillatory spectral measures of sleep stages in humans. Front Neuroinformatics 16.

Shaw P, Kabani NJ, Lerch JP, Eckstrand K, Lenroot R, Gogtay N, Greenstein D, Clasen L, Evans A, Rapoport JL, Giedd JN, Wise SP (2008) Neurodevelopmental trajectories of the human cerebral cortex. J Neurosci Off J Soc Neurosci 28:3586–3594.

Shaw P, Lerch J, Greenstein D, Sharp W, Clasen L, Evans A, Giedd J, Castellanos FX, Rapoport J (2006) Longitudinal Mapping of Cortical Thickness and Clinical Outcome in Children and Adolescents With Attention-Deficit/Hyperactivity Disorder. Arch Gen Psychiatry 63:540–549.

Shekleton JA, Rajaratnam SMW, Gooley JJ, Van RE, Czeisler CA, Lockley SW (2013) Improved Neurobehavioral Performance during the Wake Maintenance Zone. J Clin Sleep Med 09:353–362.

Sherman MA, Lee S, Law R, Haegens S, Thorn CA, Hämäläinen MS, Moore CI, Jones SR (2016) Neural mechanisms of transient neocortical beta rhythms: Converging evidence from humans, computational modeling, monkeys, and mice. Proc Natl Acad Sci 113:E4885–E4894.

Shin H, Law R, Tsutsui S, Moore CI, Jones SR (2017) The rate of transient beta frequency events predicts behavior across tasks and species. eLife 6:e29086.

Smith JR (1938) The Electroencephalogram During Normal Infancy and Childhood: I. Rhythmic Activities Present in the Neonate and Their Subsequent Development. Pedagog Semin J Genet Psychol 53:431–453.

Snipes S (2025) Iota oscillations (25-35 Hz) during wake and REM sleep in children and young adults. J Neurophysiol 134:1–9.

Snipes S, Krugliakova E, Meier E, Huber R (2022) The theta paradox: 4-8 Hz EEG oscillations reflect both sleep pressure and cognitive control. J Neurosci.

Snipes S, Meier E, Accascina S, Huber R (2024) Extended wakefulness alters the relationship between EEG oscillations and performance in a sustained attention task. J Sleep Res n/a:e14230.

Snipes S, Meier E, Meissner SN, Landolt H-P, Huber R (2023) How and when EEG reflects changes in neuronal connectivity due to time awake. iScience 26.

Somsen RJM, van’t Klooster BJ, van der Molen MW, van Leeuwen HMP, Licht R (1997) Growth spurts in brain maturation during middle childhood as indexed by EEG power spectra. Biol Psychol 44:187–209.

Steiger JH (1980) Tests for comparing elements of a correlation matrix. Psychol Bull 87:245–251.

Stroganova TA, Orekhova EV, Posikera IN (1999) EEG alpha rhythm in infants. Clin Neurophysiol 110:997–1012.

Strogatz SH, Kronauer RE, Czeisler CA (1987) Circadian pacemaker interferes with sleep onset at specific times each day: Role in insomnia. Am J Physiol - Regul Integr Comp Physiol 253.

Tal I, Neymotin S, Bickel S, Lakatos P, Schroeder CE (2020) Oscillatory Bursting as a Mechanism for Temporal Coupling and Information Coding. Front Comput Neurosci 14.

Tononi G, Cirelli C (2014) Sleep and the Price of Plasticity: From Synaptic and Cellular Homeostasis to Memory Consolidation and Integration. Neuron 81:12–34.

Tononi G, Cirelli C (2003) Sleep and synaptic homeostasis: a hypothesis. Brain Res Bull 62:143–150.

Tran Y, Craig A, Craig R, Chai R, Nguyen H (2020) The influence of mental fatigue on brain activity: Evidence from a systematic review with meta-analyses. Psychophysiology 57:e13554.

Tröndle M, Popov T, Dziemian S, Langer N (2022) Decomposing the role of alpha oscillations during brain maturation. eLife 11:e77571.

van Ede F, Quinn AJ, Woolrich MW, Nobre AC (2018) Neural Oscillations: Sustained Rhythms or Transient Burst-Events? Trends Neurosci 41:415–417.

Volk C, Jaramillo V, Merki R, O’Gorman Tuura R, Huber R (2018) Diurnal changes in glutamate + glutamine levels of healthy young adults assessed by proton magnetic resonance spectroscopy. Hum Brain Mapp 39:3984–3992.

Volk C, Jaramillo V, Studler M, Furrer M, O’Gorman Tuura RL, Huber R (2019) Diurnal changes in human brain glutamate + glutamine levels in the course of development and their relationship to sleep. NeuroImage 196:269–275.

Wascher E, Rasch B, Sänger J, Hoffmann S, Schneider D, Rinkenauer G, Heuer H, Gutberlet I (2014) Frontal theta activity reflects distinct aspects of mental fatigue. Biol Psychol 96:57–65.

Wessel JR (2020) β-Bursts Reveal the Trial-to-Trial Dynamics of Movement Initiation and Cancellation. J Neurosci 40:411–423.

Wilhelm I, Kurth S, Ringli M, Mouthon A-L, Buchmann A, Geiger A, Jenni OG, Huber R (2014) Sleep Slow-Wave Activity Reveals Developmental Changes in Experience-Dependent Plasticity. J Neurosci 34:12568–12575.

Wilkinson CL, Yankowitz LD, Chao JY, Gutiérrez R, Rhoades JL, Shinnar S, Purdon PL, Nelson CA (2024) Developmental trajectories of EEG aperiodic and periodic components in children 2–44 months of age. Nat Commun 15:5788.

Xian P, Sheng X, Liu S, Liu Z, Guo X (2025) Sleep dysregulation in ADHD children: a systematic review and meta-analysis. Psychol Med 55:e321.

Zich C, Quinn AJ, Mardell LC, Ward NS, Bestmann S (2020) Dissecting Transient Burst Events. Trends Cogn Sci 24:784–788.

Zimmermann P, Fimm B (2012) Testbatterie zur Erfassung von Aufmerksamkeitsstörungen (TAP 2.3.).
