# Supplementary material for: The Interaction between Sleep and Development on Wake EEG Oscillations
Source: eNeuro. 2026 Apr 21;13(4):ENEURO.0384-25.2026. doi: 10.1523/ENEURO.0384-25.2026 (PMC13132015; doi:10.1523/ENEURO.0384-25.2026)
Supplement: Data 1 — MATLAB code used to preprocess, analyze and plot the wake EEG data used throughout the manuscript. Includes the primary repository children-wake/ as well as the required toolboxes: chART, fooof_mat, & Matcycle. Download Data 1, ZIP file. [file eneuro-13-ENEURO.0384-25.2026-s006.zip › Code/toolboxes/Matcycle/docs/Cycle by Cycle Analysis.pdf]

Cycle by Cycle Analysis

Workshop

10.08.23

By Sophia Snipes

Schedule

2:00-3:00	Presentation on how cycle-by-cycle analysis works
3:00-3:30	Coffee break
3:30-5:30	Practical part
5:30-??	Apero!

Cycle-by-cycle analysis is for detecting oscillation bursts

Cycle-by-cycle analysis finds bursts of oscillations in the EEG so that they can be quantified based on their:

- Amplitudes
- Frequency
- Durations
- Spatial distribution
- Shape

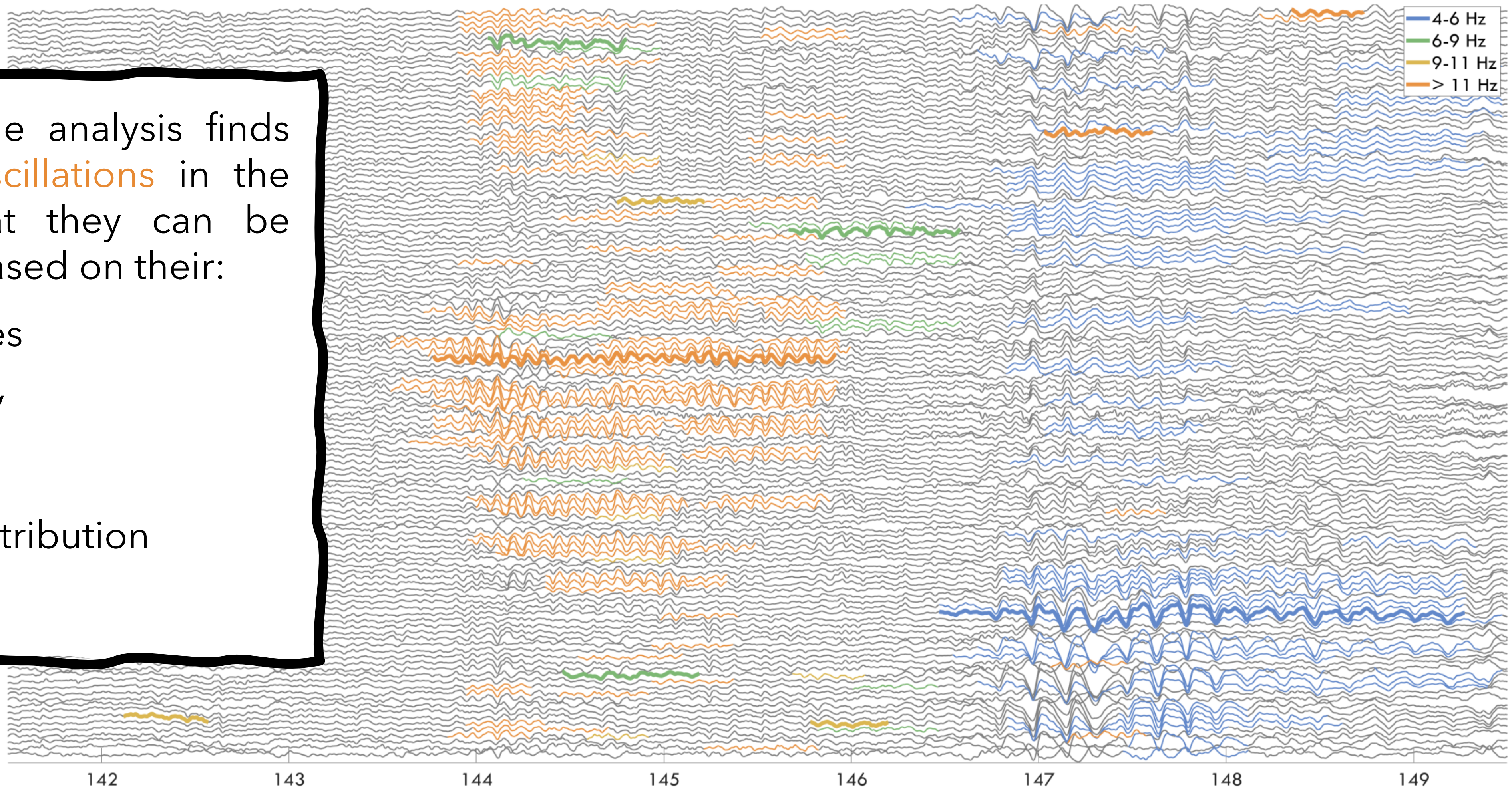

The alternatives for quantifying oscillations

Spectral power

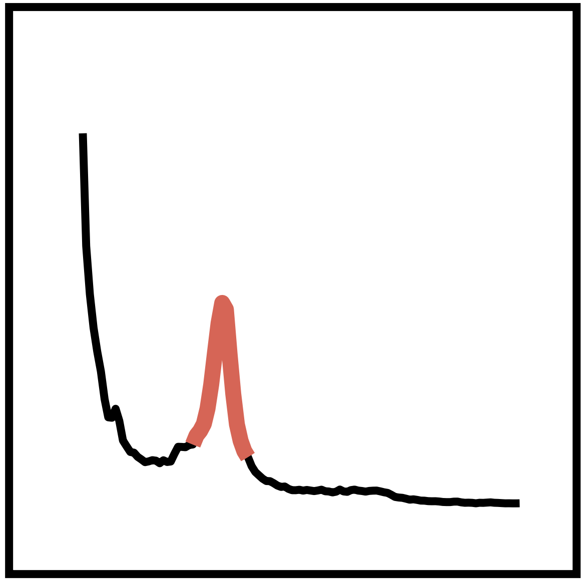

FOOOF

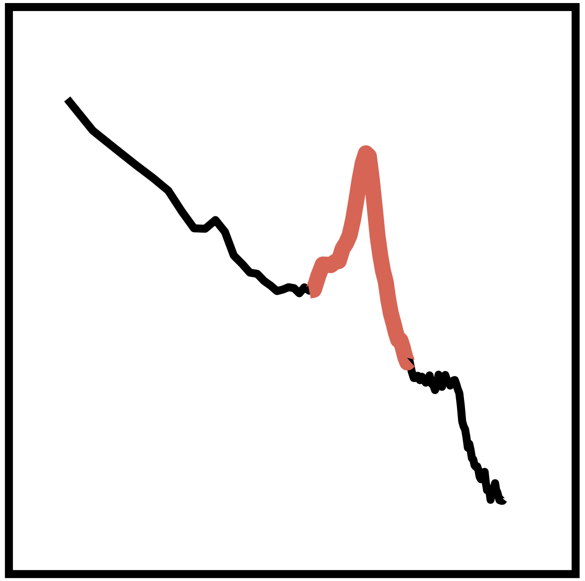

Time-frequency analysis

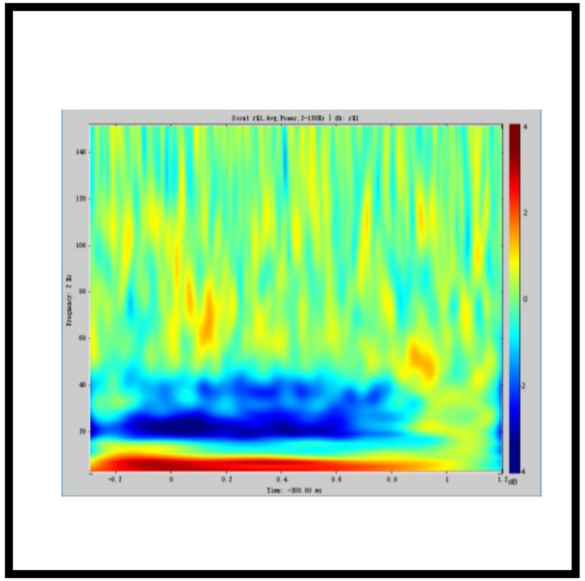

Thresholds

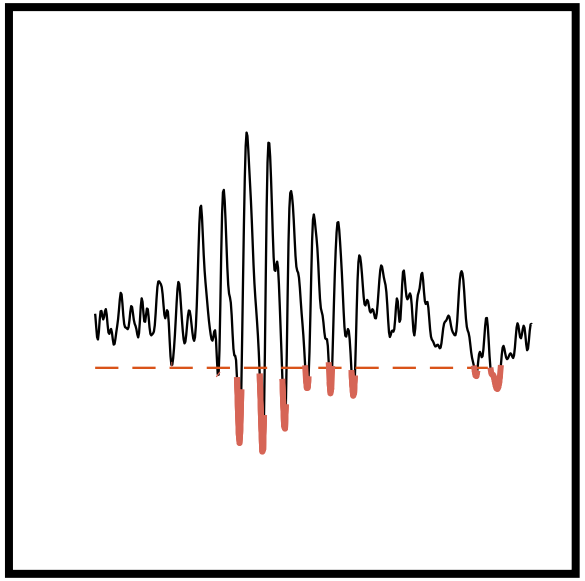

Problem 1: the EEG is non-stationary

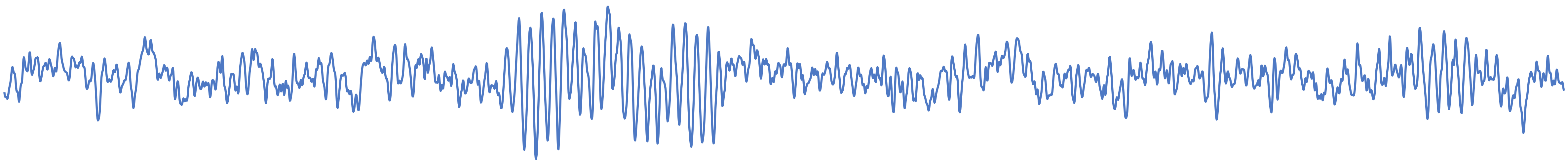

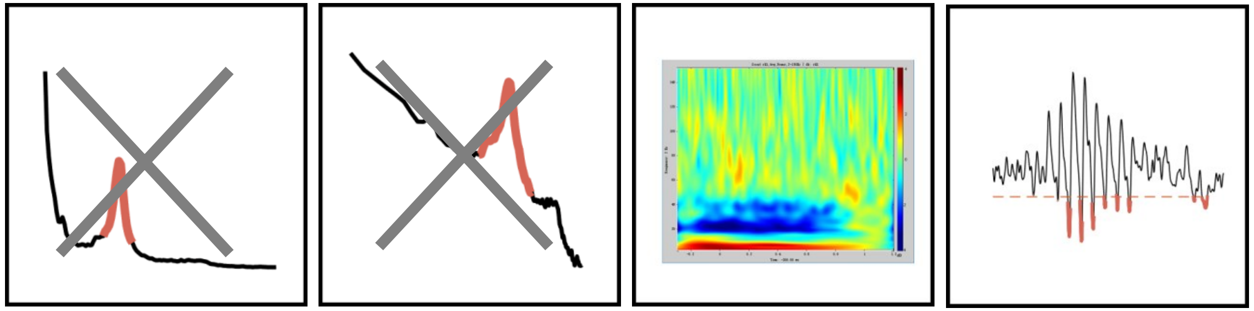

EEG oscillation bursts come and go, so simple power is not sufficient to quantify changes

Problem 2: EEG is made of both oscillations and “colored background noise”

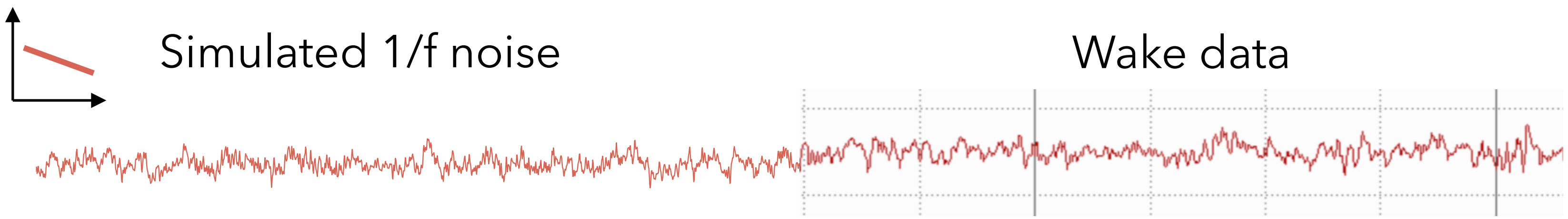

More than white noise, colored noise makes it all too easy to find false positive oscillations in the signal.

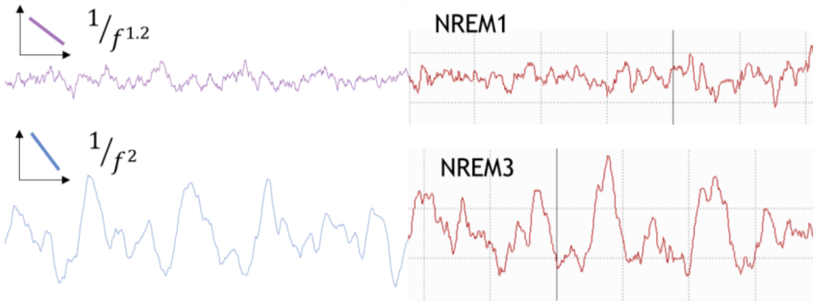

Background also changes with brain state, independently from oscillations.

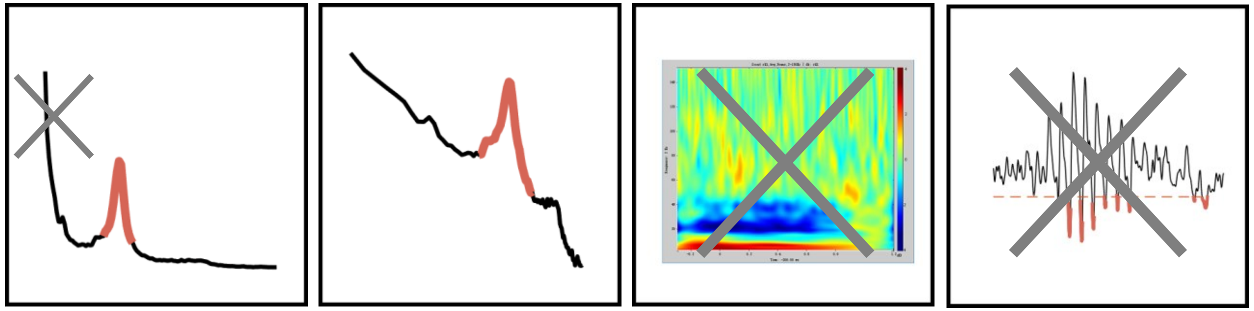

Donoghue et al., 2020

Solution: cycle-by-cycle analysis finds bursts based on the shape of the signal

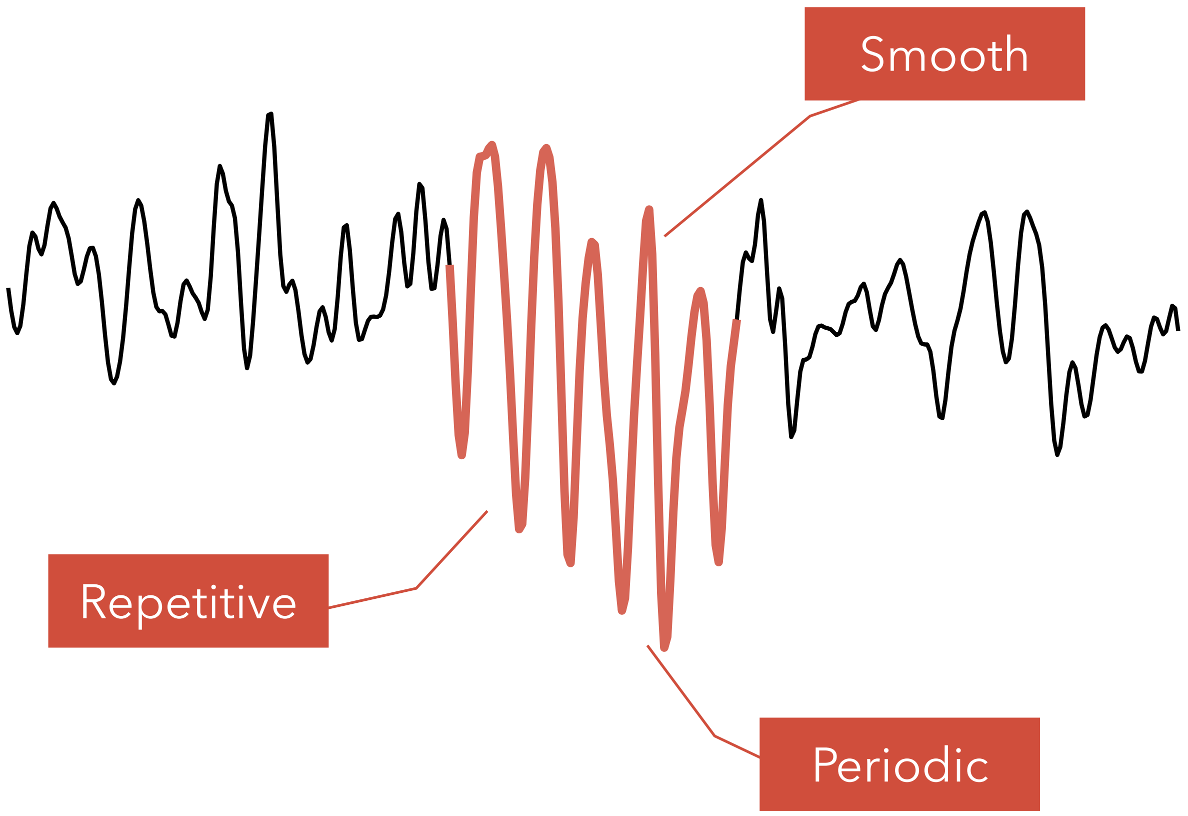

Other advantages:

- High temporal resolution
- High frequency resolution
- Robust to non-sinusoidal oscillations

Cole & Voytek, 2019

What can you do with cycle-by-cycle analysis?

- Cross-frequency coupling
- Connectivity analysis
- Burst categorization by waveform
- Dissociate changes in amplitude from quantities of oscillations
- Travelling waves
- Infra-slow oscillations

If it involves oscillations, then this is the analysis for you!

When NOT to use cycle-by-cycle analysis?

When interested in the background activity

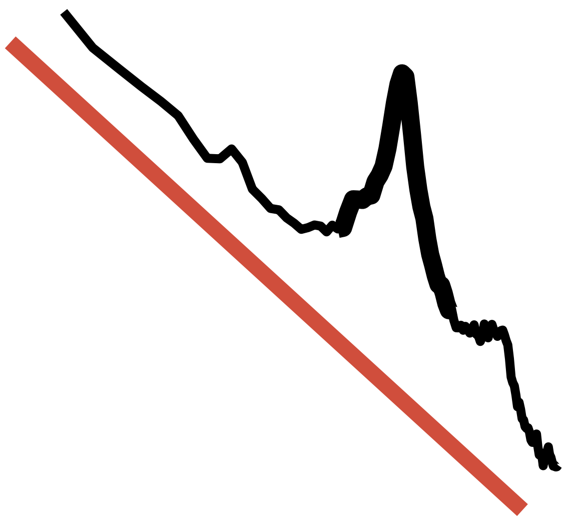

When interested in isolated waves

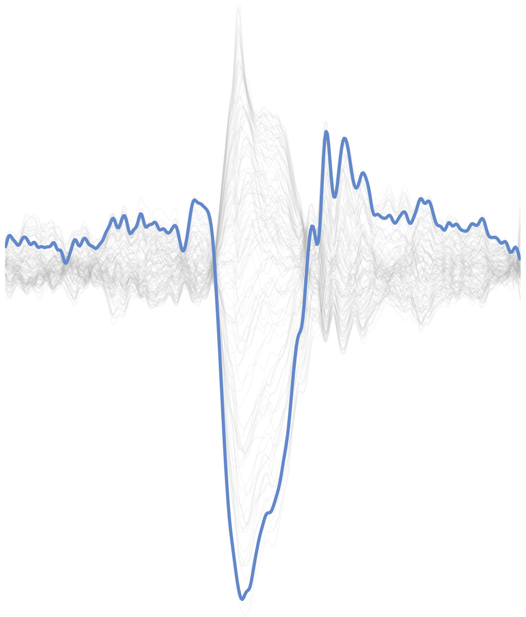

Certain cross-frequency coupling

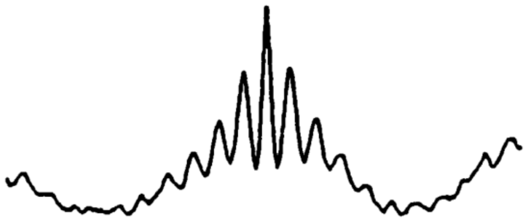

How it works

Step 1: Filter EEG data

Step 2: Detect all the cycles

Step 3: Group periodic cycles

Step 1: Filtering EEG data

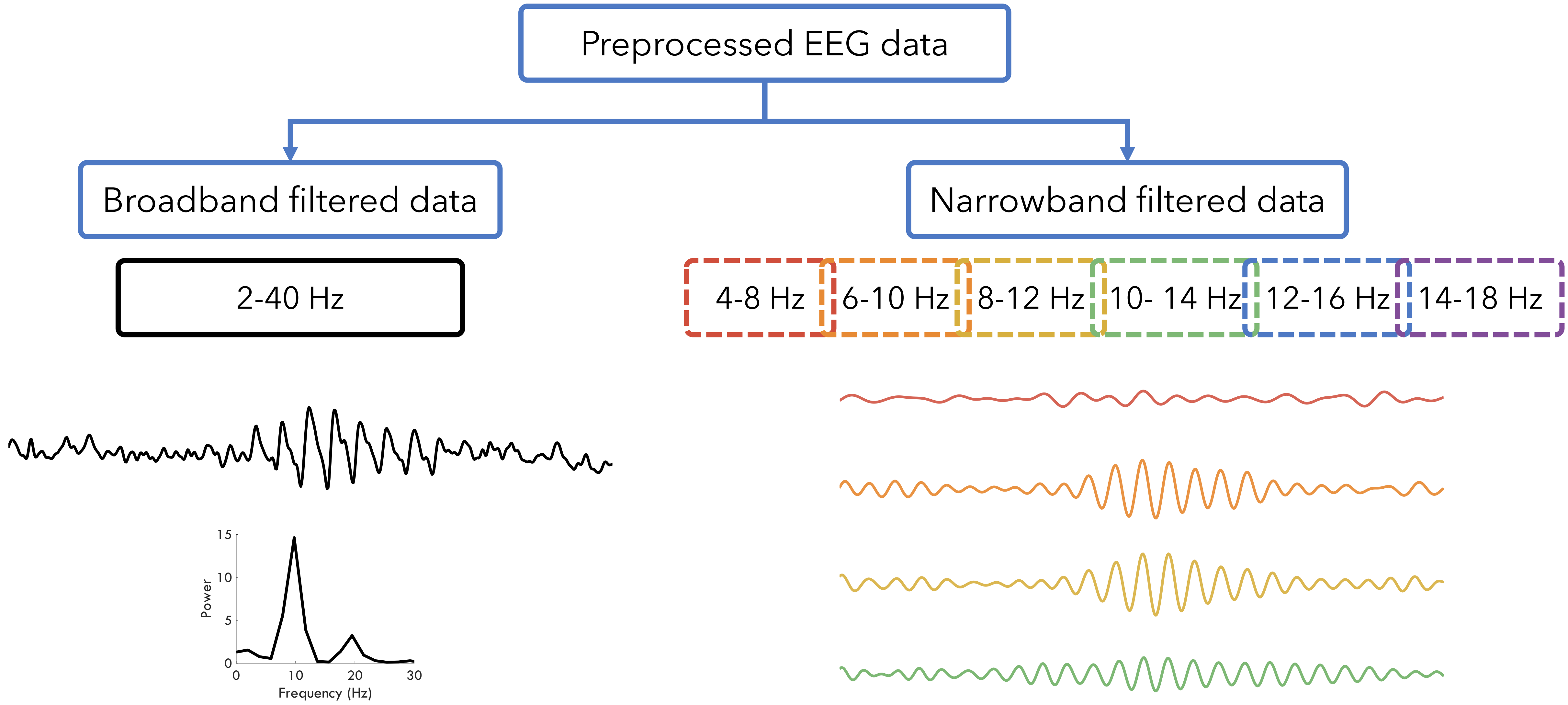

Step 2: detect all the cycles

I: Detect zero-crossings

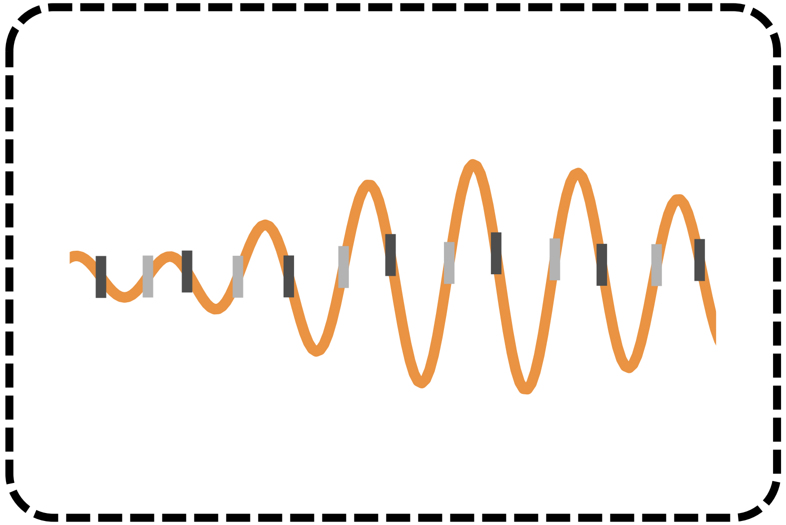

In **narrowband** data, identify all the positive and negative zero-crossings. N.B. this is the only step on the narrowband data.

II: Detect peaks

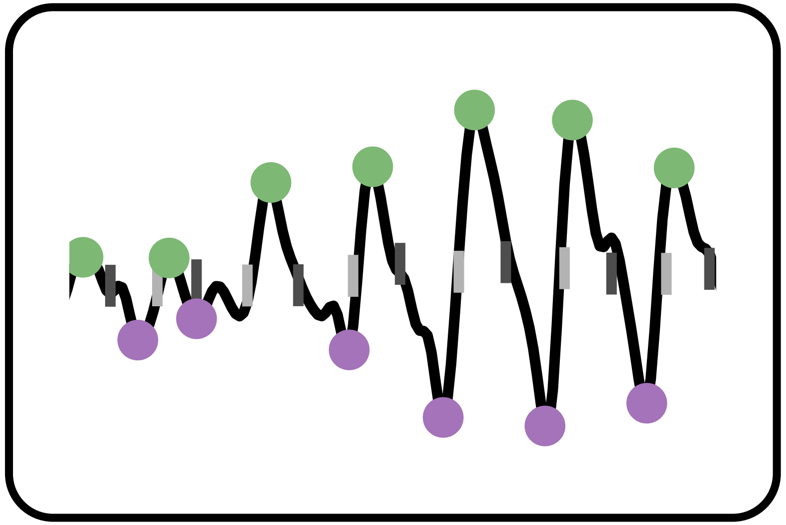

In the **broadband** data, detect positive and negative peaks between zero-crossings (max absolute value).

III: Divide into cycles

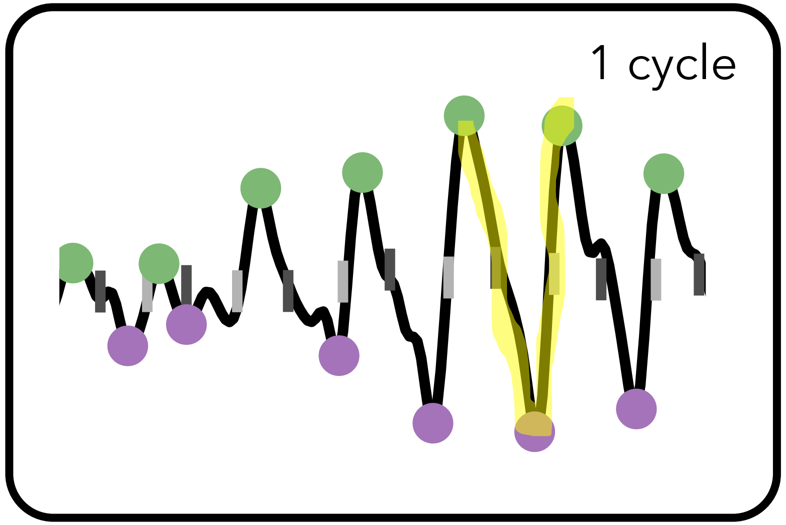

Each cycle is referenced to the negative peak, and starts and ends with the two neighboring positive peaks.

Step 3: group periodic cycles into bursts

I:
Assign properties to each cycle

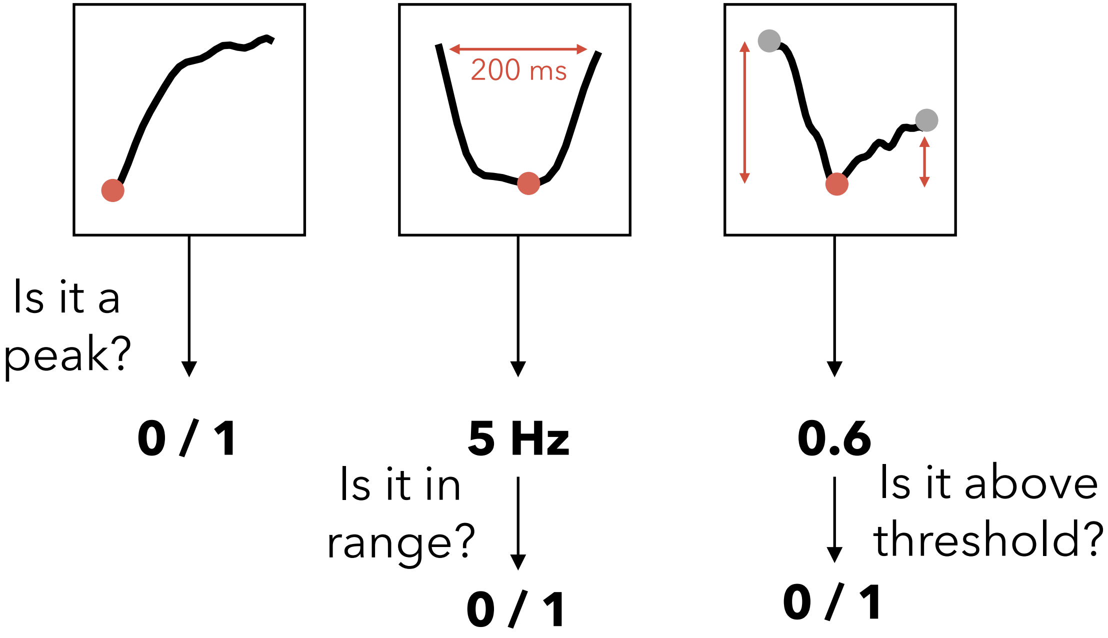

II:
Bursts are when at least N cycles in a row meet all the criteria

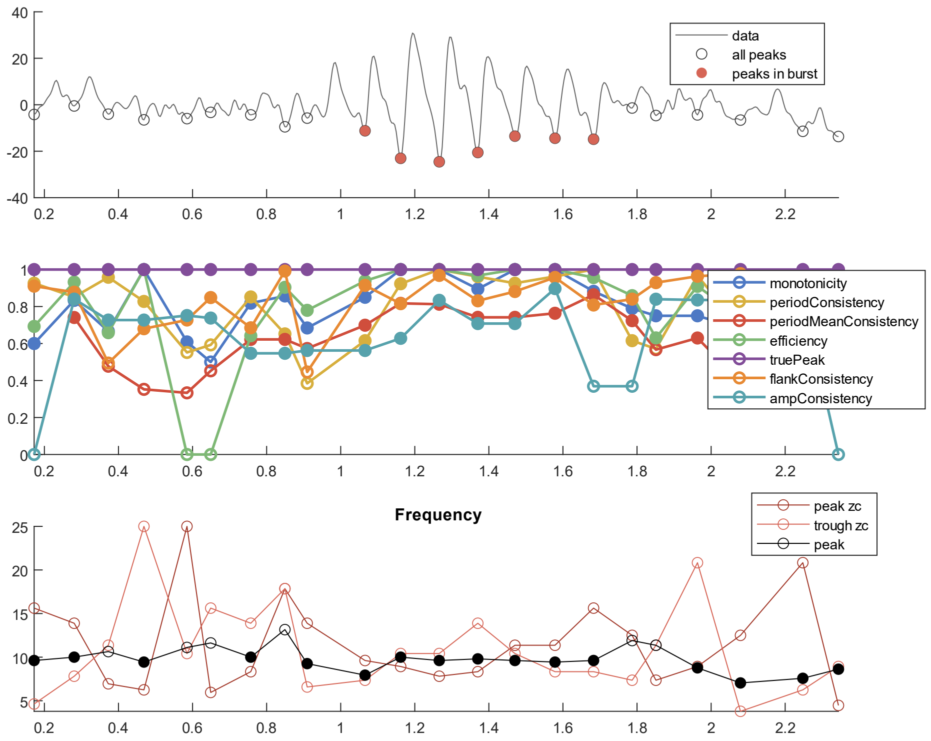

For best results, apply multiple passes of burst detection

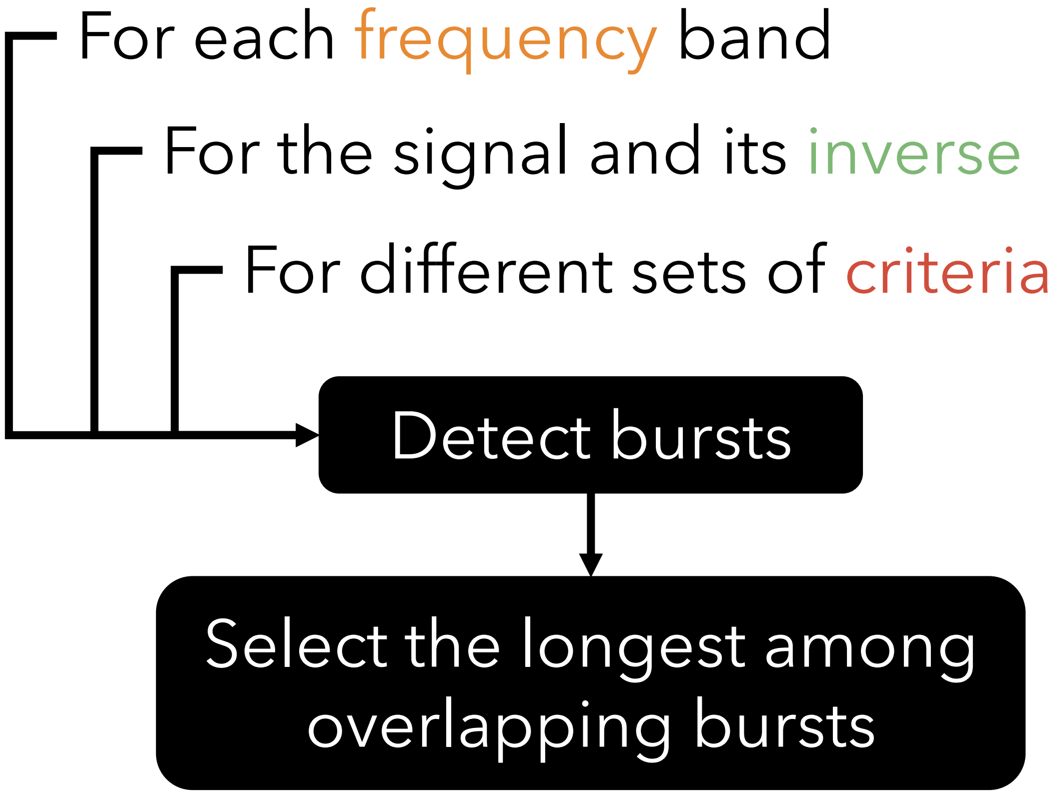

Criteria

Local minimum

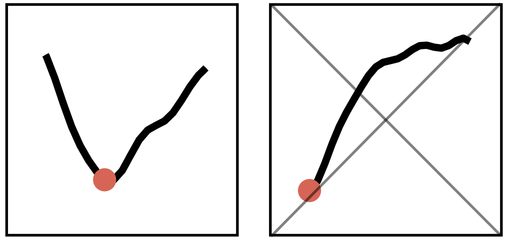

Correct period (per band)

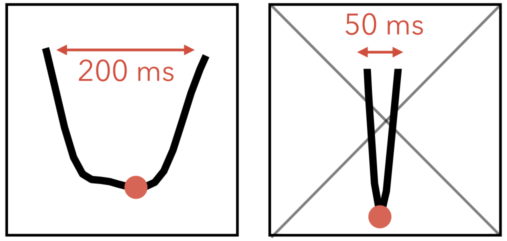

Consistent period

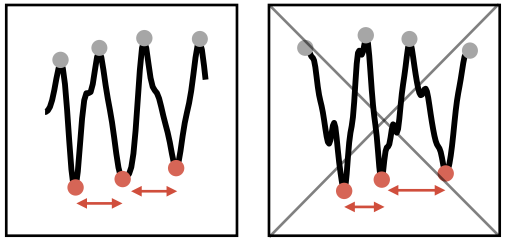

Consistent flank amplitudes

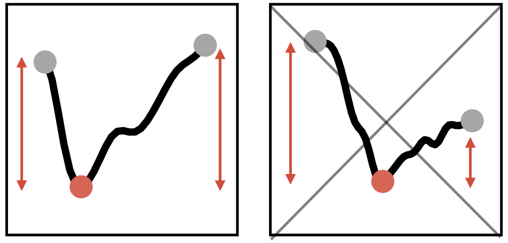

Consistent peak amplitudes

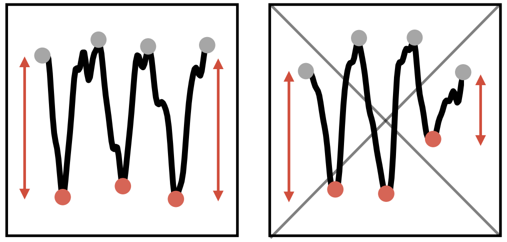

Monotonicity

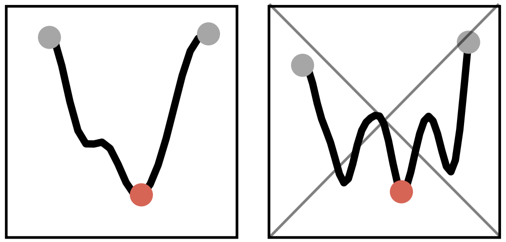

NOT Criteria

Minimum amplitude

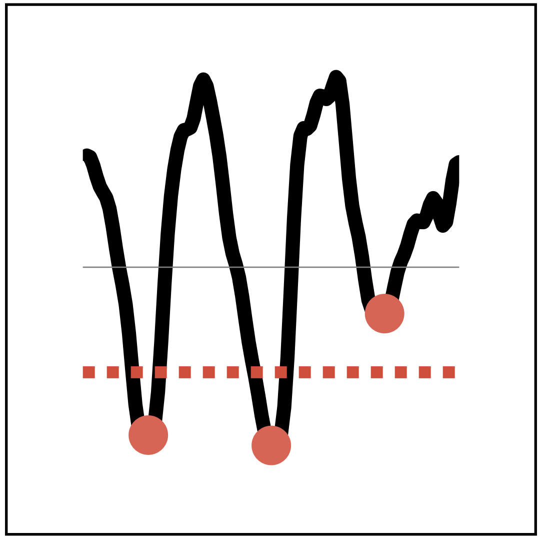

The nitty-gritty details

Glossary

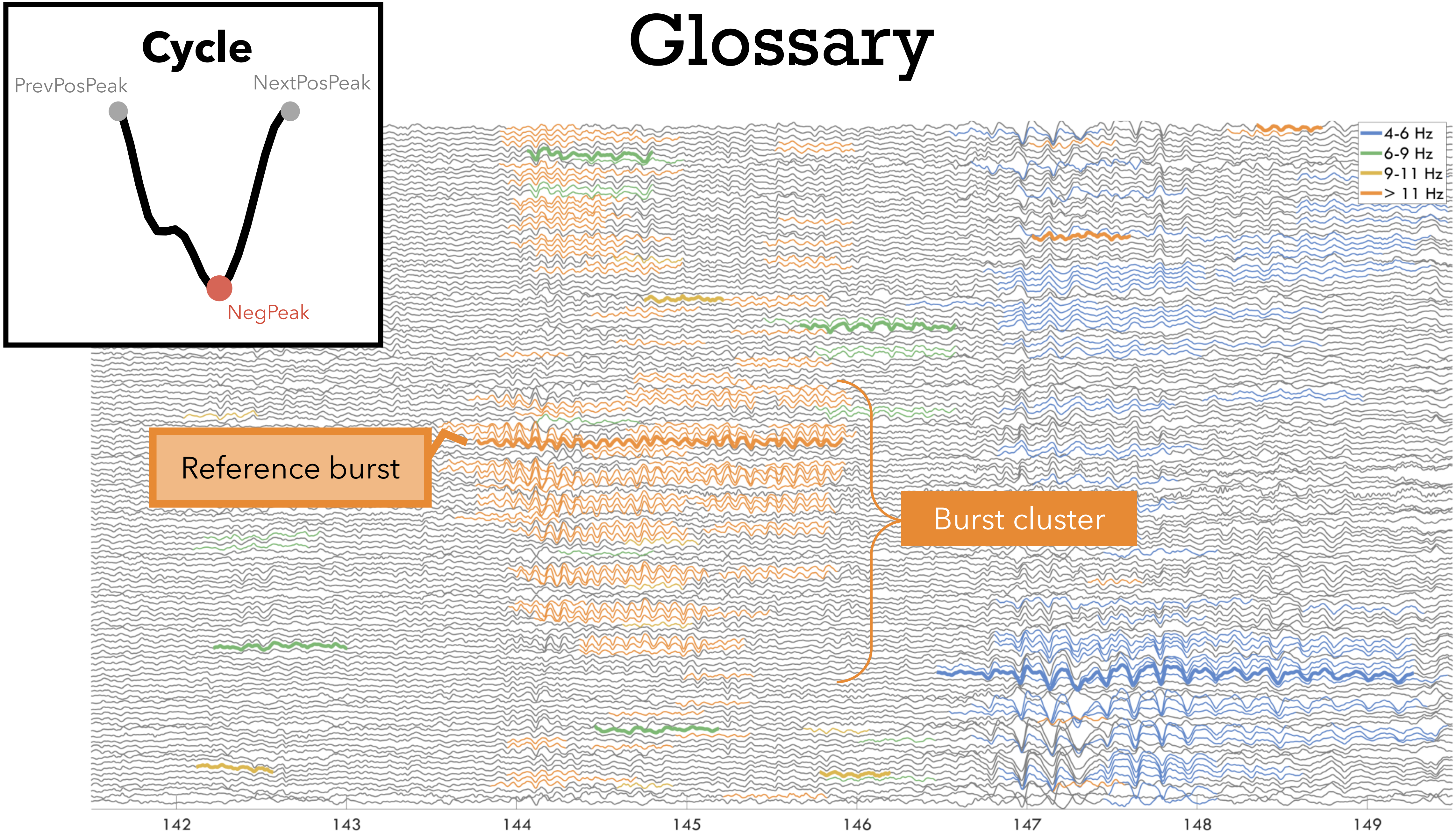
Cycle
PrevPosPeak
NextPosPeak
NegPeak
4-6 Hz
6-9 Hz
9-11 Hz
> 11 Hz
Reference burst
Burst cluster
142
143
144
145
146
147
148
149

The process

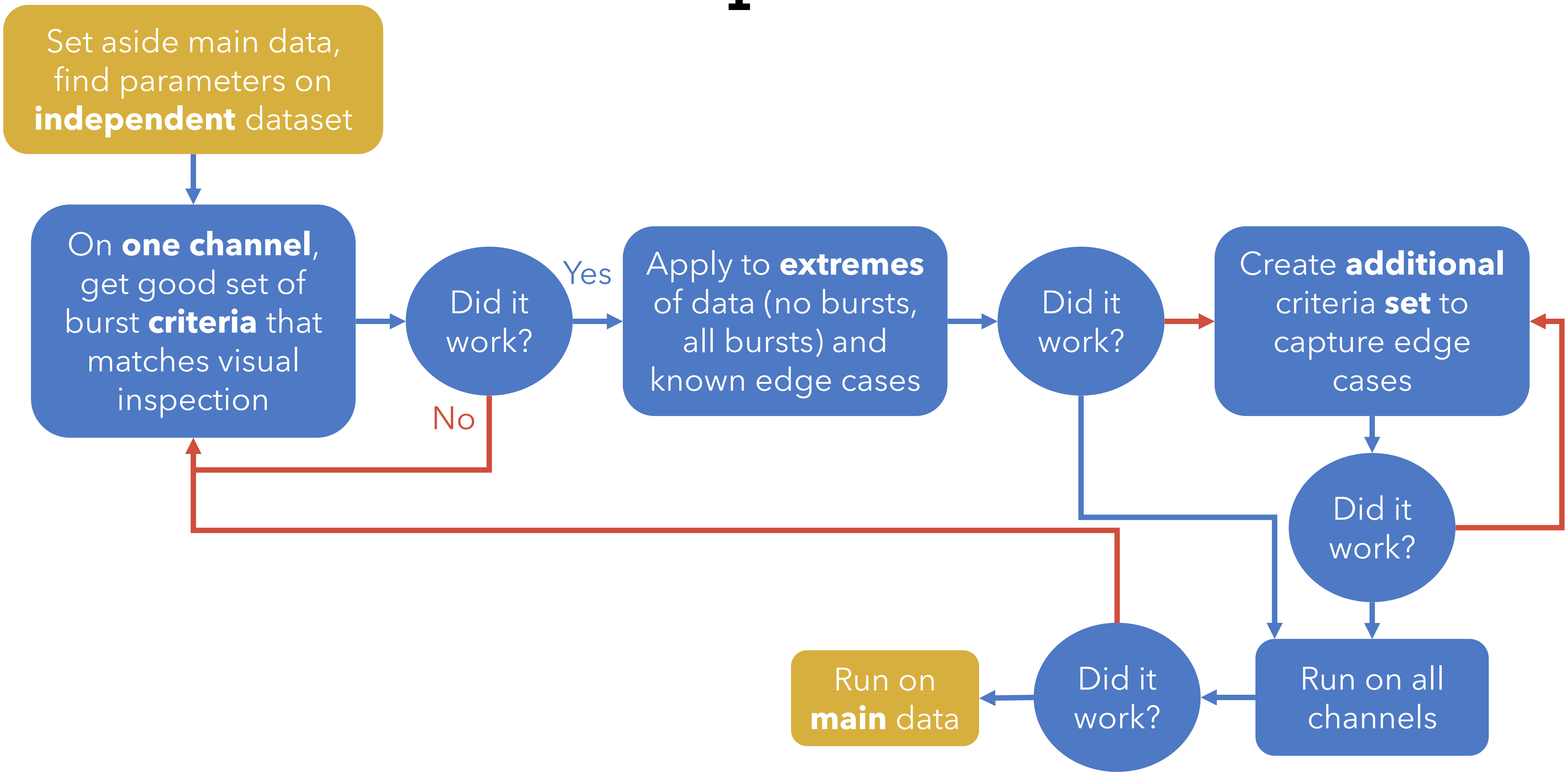
Set aside main data, find parameters on **independent** dataset
On **one channel**, get good set of burst **criteria** that matches visual inspection
Did it work?
Yes
No
Apply to **extremes** of data (no bursts, all bursts) and known edge cases
Did it work?
Create **additional** criteria **set** to capture edge cases
Did it work?
Run on all channels
Did it work?
Run on **main** data

The repository

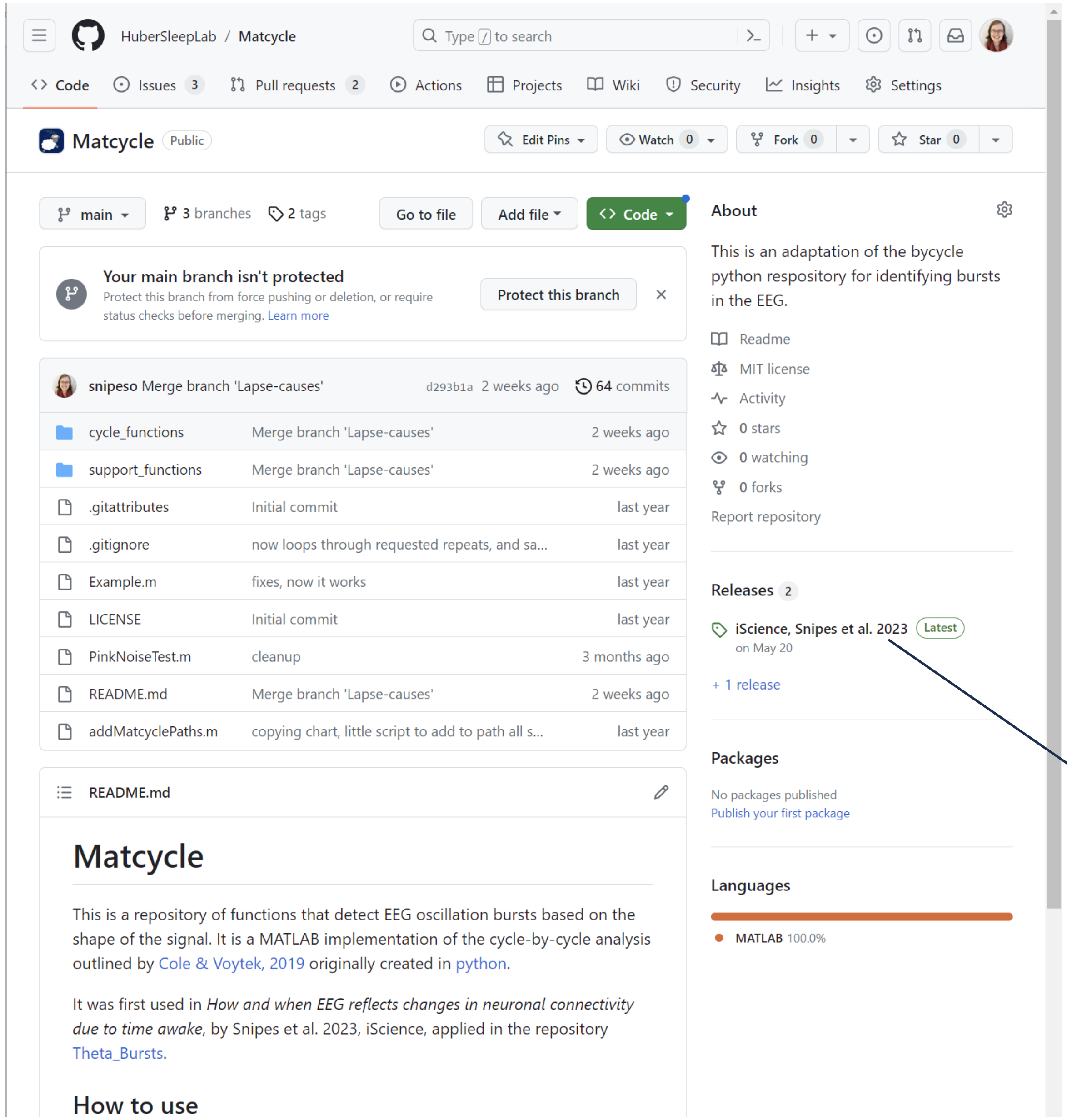

https://github.com/HuberSleepLab/Matcycle

When publishing, create a “release”, so you immortalize the version actually used to obtain results

+Packages in MATLAB

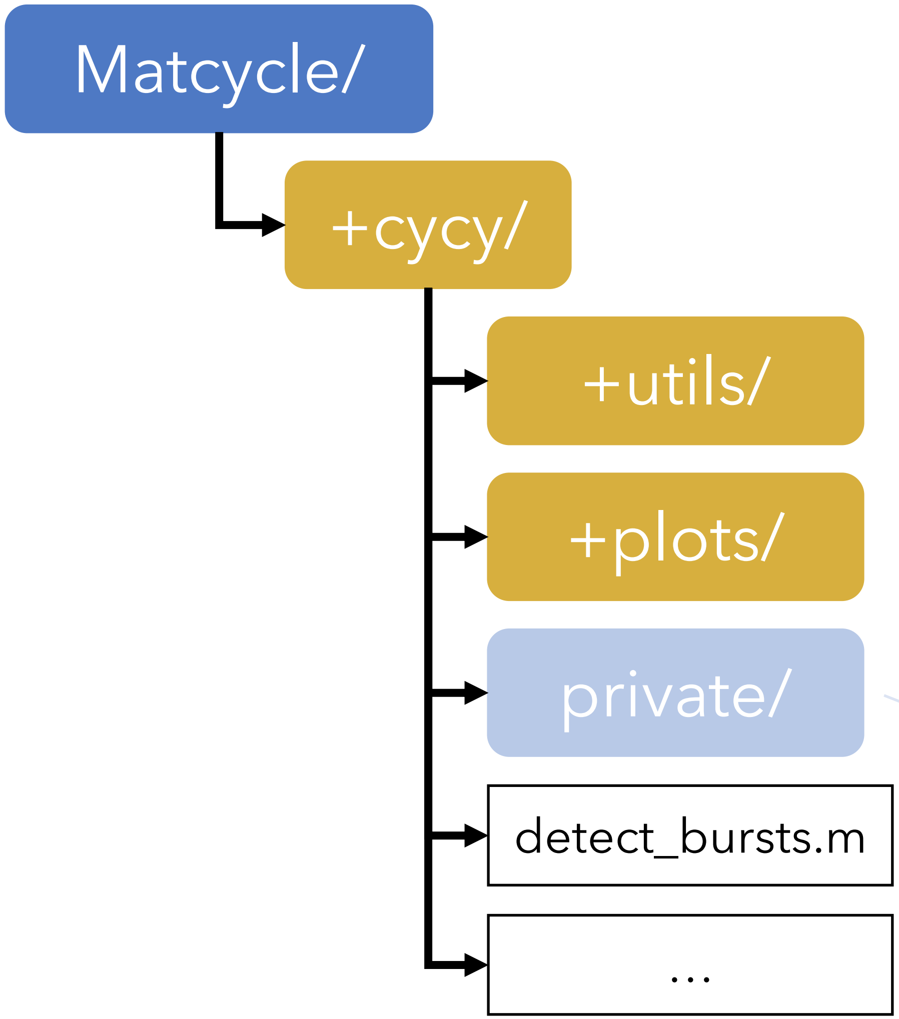

1) Add the Matcycle folder to path
2) Call the main functions like so:
   "`cycy.detect_bursts()`"
3) Call support functions like so:
   "`cycy.utils.highpass_filter()`"

In MATLAB, if you have a folder called "private", the functions can be called by scripts within the folder above, but not by scripts outside

The code

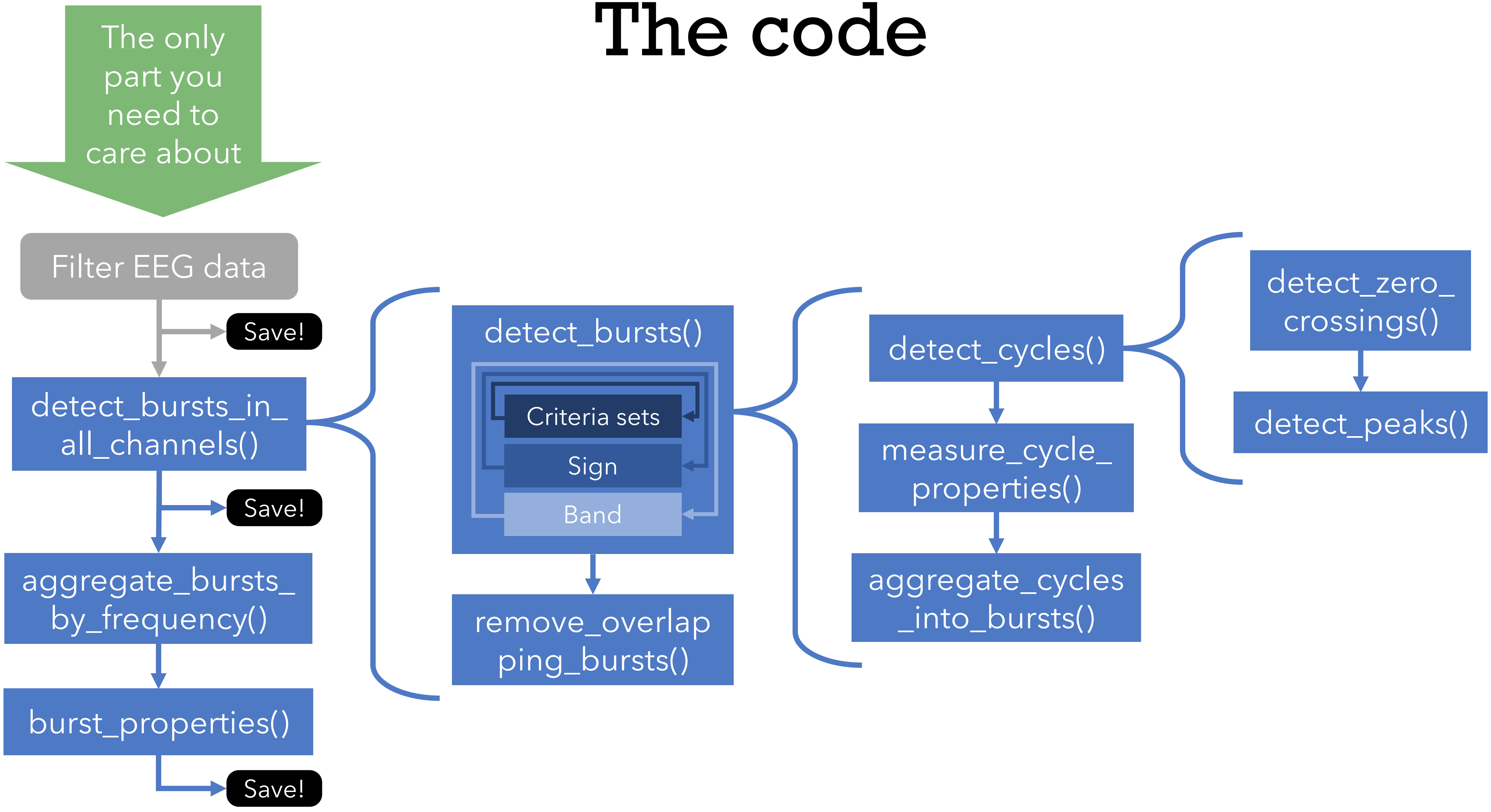
The only part you need to care about
Filter EEG data
Save!
detect_bursts_in_all_channels()
Save!
aggregate_bursts_by_frequency()
burst_properties()
Save!
detect_bursts()
Criteria sets
Sign
Band
remove_overlapping_bursts()
detect_cycles()
measure_cycle_properties()
aggregate_cycles_into_bursts()
detect_zero_crossings()
detect_peaks()

The Inputs

Criteria sets

```
>> CriteriaSets

CriteriaSets =

  1×2 struct array with fields:

    isTruePeak
    PeaksCount
    FlankConsistency
    MonotonicityInTime
    MonotonicityInAmplitude
    isProminent
    PeriodConsistency
    AmplitudeConsistency
    MinCyclesPerBurst
    VoltageNeg
    Amplitude
```

EEG data

```
>> EEGbroadband

EEGbroadband =

  struct with fields:

       srate: 250
        data: [123×7501 single]
    chanlocs: [1×123 struct]

>> EEGnarrowbands

EEGnarrowbands =

  1×4 struct array with fields:

    srate
    data
    chanlocs
```

The Output: Cycles

Cycle Detection

```
>> Cycles

Cycles =

  1×3381 struct array with fields:

    NegPeakIdx
    PrevPosPeakIdx
    NextPosPeakIdx
```

Cycle Properties

```
>> AugmentedCycles

AugmentedCycles =

  1×3379 struct array with fields:

    NegPeakIdx
    PrevPosPeakIdx
    NextPosPeakIdx
    VoltagePrevPos
    VoltageNeg
    VoltageNextPos
    isTruePeak
    PeaksCount
    PeriodPos
    PeriodNeg
    Frequency
    Amplitude
    AmplitudeRamp
    FlankConsistency
    MonotonicityInTime
    MonotonicityInAmplitude
    isProminent
    PeriodConsistency
    AmplitudeConsistency
```

The Output: Bursts

Bursts

```
>> Bursts

Bursts =

  1×437 struct array with fields:

    CyclesCount
    CycleIndexes
    NegPeakIdx
    PrevPosPeakIdx
    NextPosPeakIdx
    VoltagePrevPos
    VoltageNeg
    VoltageNextPos
    isTruePeak
    PeaksCount
    PeriodPos
    PeriodNeg
    Frequency
    Amplitude
    AmplitudeRamp
    FlankConsistency
    MonotonicityInTime
    MonotonicityInAmplitude
    isProminent
    PeriodConsistency
    AmplitudeConsistency
    Start
    End
    BurstFrequency
    Band
    ChannelIndex
    ChannelIndexLabel
    Sign
    CriteriaSetIndex
```

Burst Cluster

```
>> BurstClusters

BurstClusters =

  1×53 struct array with fields:

    CyclesCount
    CycleIndexes
    NegPeakIdx
    PrevPosPeakIdx
    NextPosPeakIdx
    VoltagePrevPos
    VoltageNeg
    VoltageNextPos
    isTruePeak
    PeaksCount
    PeriodPos
    …
    ChannelIndexLabel
    Sign
    CriteriaSetIndex
    ClusterBurstsIdx
    ClusterChannelIndexes
    ClusterChannelLabels
    ClusterStarts
    ClusterEnds
    ClusterCycleCounts
    ClusterSigns
    ClusterFrequency
    ClusterAmplitude
    ClusterAmplitudeSum
    ClusterPeaks
    ClusterStart
    ClusterEnd
    ClusterGlobality
```

Cycle information of reference burst

How to chose criteria and thresholds?

Does it correctly capture obvious bursts?

Why did it miss this burst?

Does it correctly reject background activity?

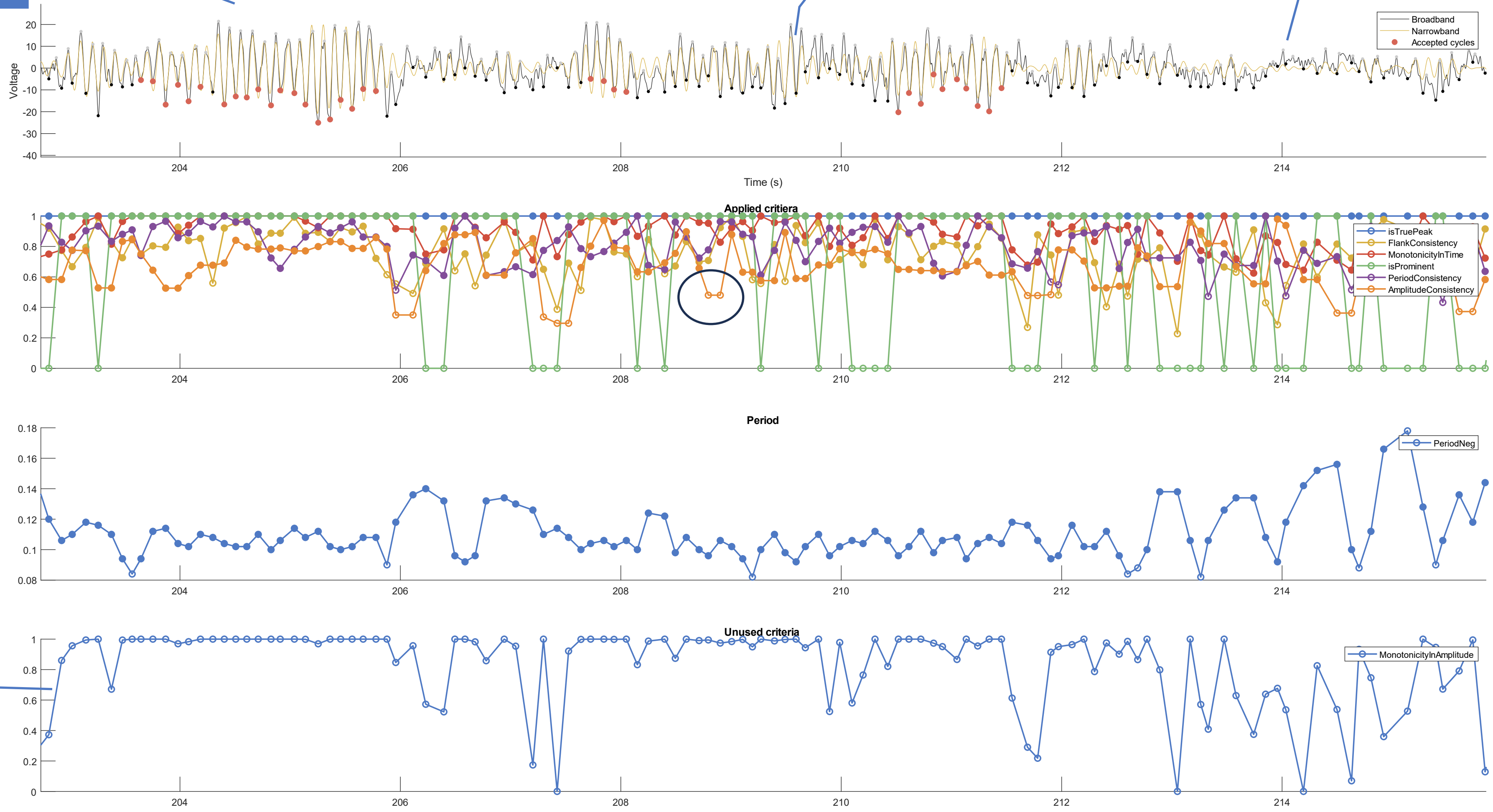

Was a criteria I didn't use better?

Diagnostics: which criteria were more aggressive?

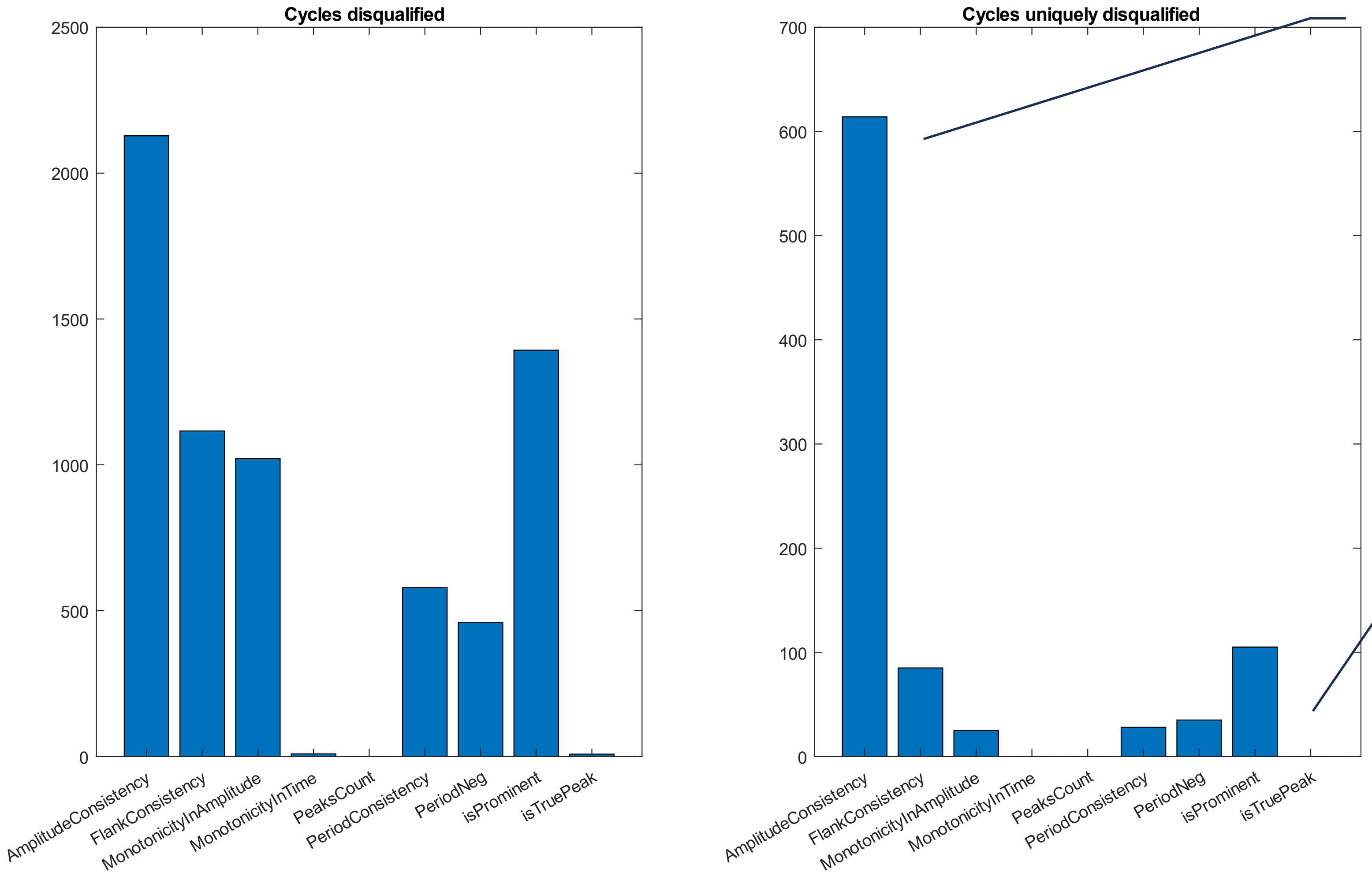

If one criteria is solely responsible for disqualifying a lot of cycles, threshold might be too strict

Could have omitted

Diagnostics: properties distributions

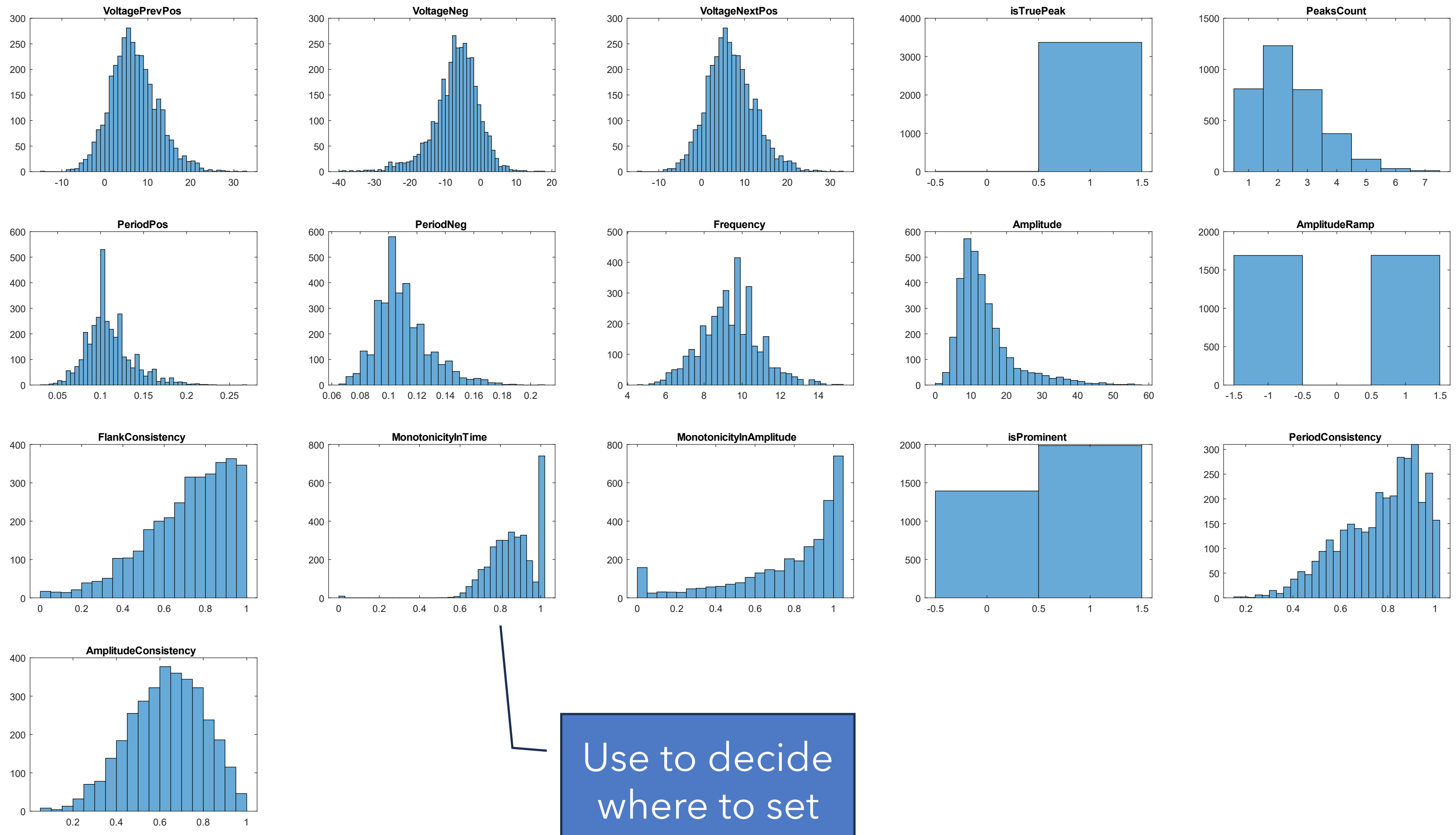

Use to decide where to set thresholds

Practical Part

Recommended Steps

1. Download the repository: https://github.com/HuberSleepLab/Matcycle
2. Run **SingleBandExample.m**. To understand how the analysis works, try adapting:
    - The EEG channel (if you have it, try with your own data!)
    - The narrow band frequency range
    - Criteria set thresholds
3. Run & adapt **MultiChannelExample.m** for your own data
4. Exercise for home: how long does it take to fall asleep?
    - Automatically quantify the interval between the last alpha burst and the first sleep spindle for your data

Bibliography

- Donoghue, T., Haller, M., Peterson, E. J., Varma, P., Sebastian, P., Gao, R., ... & Voytek, B. (2020). Parameterizing neural power spectra into periodic and aperiodic components. *Nature neuroscience*, *23*(12), 1655-1665.
- Cole, S., & Voytek, B. (2019). Cycle-by-cycle analysis of neural oscillations. *Journal of neurophysiology*, *122*(2), 849-861.
- Snipes, S., Meier, E., Meissner, S. N., Landolt, H. P., & Huber, R. (2023). How and when EEG reflects changes in neuronal connectivity due to time awake. *iScience*.
